# Melatonin modulates the hypothalamic-pituitary neuroendocrine axis to regulate physiological color change in teleost fish

**DOI:** 10.7150/ijbs.81055

**Published:** 2023-06-04

**Authors:** Jiaqian Feng, Jingwen Yang, Zhijing Jiang, Naiming Zhou, Xue Liu, Guangbo Zhang, Xiaojun Yan, Jixiu Wang, Xiuwen Xu, Su Guo, Tianming Wang

**Affiliations:** 1 National Engineering Laboratory of Marine Germplasm Resources Exploration and Utilization, Marine Science College, Zhejiang Ocean University, Zhoushan, Zhejiang 316022, People's Republic of China.; 2 Institute of Biochemistry, College of Life Sciences, Zijingang Campus, Zhejiang University, Hangzhou, Zhejiang 310058, People's Republic of China.; 3 Programs in Human Genetics and Biological Sciences, Department of Bioengineering and Therapeutic Sciences, University of California, San Francisco, San Francisco, CA 94158, United States.

**Keywords:** melatonin receptors, cell signaling, single-cell RNA-seq, hypothalamic-pituitary axis, chromatophores

## Abstract

Melatonin (MT) is a crucial neuroendocrine regulator of various physiological activities in vertebrates, especially in circadian or seasonal rhythm control. In the present study, the large yellow croaker (*Larimichthys crocea*), a marine bony fish with circadian body color change behavior, is chosen for functional investigation on teleost MT signaling systems that remain uncharacterized. All five melatonin receptors (*Lc*Mtnr1a1, *Lc*Mtnr1a2, *Lc*Mtnr1b1, *Lc*Mtnr1b2, and *Lc*Mtnr1c) were significantly activated by MT, triggering extracellular signal-regulated kinase 1/2 (ERK1/2) phosphorylation through different G protein coupling signaling pathways, with exclusive G_αi_-dependency for *Lc*Mtnr1a2 and *Lc*Mtnr1c, and G_αq_-dependency for two *Lc*Mtnr1b paralogs, whereas *Lc*Mtnr1a1 activated G_αi_ and G_αs_ dual-dependent signaling pathways. A comprehensive model of the MT signaling system in the hypothalamic-pituitary neuroendocrine axis was further constructed based on ligand-receptor interaction analysis using single-cell RNA-seq data, as well as spatial expression patterns of Mtnrs and related neuropeptides in central neuroendocrine tissues. A novel regulatory pathway of MT/melanin-concentrating hormone (MCH) and MT/(tachykinin precursor 1 (TAC1)+corticotropin-releasing hormone (CRH))/melanocyte-stimulating hormone (MSH) was discovered that functions in chromatophore mobilization and physiological color change and was further validated by pharmacological experiments. Together, our findings define multiple intracellular signaling pathways mediated by *L. crocea* melatonin receptors and provide the first in-depth evidence that uncover the upstream modulating roles of the MT signaling system in the hypothalamic-pituitary neuroendocrine axis of a marine teleost species*,* particularly in chromatophore mobilization and physiological color change.

## Introduction

In most vertebrates, especially mammals, melatonin (MT) is primarily synthesized and secreted in a circadian manner by the pineal gland 1. It is also secreted in peripheral locations such as the gastrointestinal tract, liver, and retina 2-4. Even though the photoperiodic control of MT biosynthesis has been profoundly modified throughout evolution, the MT molecule is the same in fish and mammals, with a highly conserved circadian pattern 5, 6. Furthermore, direct effects of MT on the hypothalamus and pituitary have been well characterized in both mammals and bony fishes 7-9. Studies have shown that MT was involved in reproduction control by activating the hypothalamo-pituitary-gonadal (HPG) axis with positive or negative effects depending on the specific animal species, treatment conditions or physiological states 10-13. Besides, the functions of MT on cyclical body temperature control through hypothalamic-pituitary-thyroid (HPT) axis regulation was also verified in human 14. Therefore, MT driven by the circadian clock is one of the most prominent outputs of the circadian system as an upstream modulator of the hypothalamic-pituitary axis 15.

MT functions as a neuroendocrine hormone to regulate various physiological processes mainly through melatonin receptors (Mtnrs), which belong to the G protein-coupled receptor superfamily (GPCRs). The high-affinity Mtnrs, including the Mtnr1a and Mtnr1b (also known as MT1 and MT2, respectively), are characterized in mammals 16-18. Mtnr1c (also known as MT3) studied in birds 19, amphibia 20, and fishes 21, is functionally linked to the regulation of the neuroendocrine system. Mtnr1a and Mtnr1b in the mammalian brain have important physiological functions in sleep, anxiety, pain and circadian rhythm 22. Mtnr1c expression is detected in the nervous tissues of fish 23 and other non-mammalian species 24, and possibly has a regulatory role in the photoperiodic control of neuroendocrine functions 7, such as seasonal reproduction in birds 25.

The role of MT signaling system in the control of neuroendocrine physiology, such as metabolism, energy balance, reproduction, and particularly pigment granule mobilization, is yet to be elucidated. In the early 20^th^ century, pineal gland extract was first discovered to play an important role in melanophore aggregation in amphibians, which causes lightening of the skin color 26. However, the ability of MT to quickly lighten skin color has not been determined in mammals 27. Recent evidence suggests that melanogenesis and melanocyte proliferation in humans are inhibited by MT through stimulation of its receptors (MTNR1A and MTNR1B) 28, but the molecular mechanism of MT/MTNRs action *in vivo* is still unclear. In fish, several findings suggest that MT may regulate physiological color change through chromatophore mobilization (aggregation or dispersion) 6, 29. For instance, in *Nannostomus trifasciatus* 30, the active dispersion of melanosomes in melanophores was induced by the administration of MT and its receptors. Given that the peptidergic pathways in camouflage has been clarified in zebrafish and other bony fish species, the corticotropin-releasing hormone (CRH)/α-melanocyte-stimulating hormone (α-MSH) in chromatophore dispersion and melanin-concentrating hormone (MCH) in chromatophore aggregation, it can be speculated that MT may function together with these neuropeptides in skin coloration of fishes 31-34. However, cell type-specific MT ligand-receptor interactions in the neuroendocrine center and how such interaction modulates the mobilization of chromatophores responsible for integumentary coloration in teleosts remain unclear.

The large yellow croaker (*Larimichthys crocea*) is a commercial marine fish species inhabiting the coastal areas of East and Southeast China 35. Being a near-shore migratory and seasonally reproductive species 36, the *L. crocea* exhibits significant seasonal and circadian rhythms. One of its most important traits is its changing skin coloration (especially in the ventral area) that follows a 24-hour cycle, appearing silvery-white during daytime and golden yellow at night 31. The neuroendocrine control of the dispersion and aggregation of *L. crocea* xanthophores and melanophores is conserved, sharing the same regulatory pathway found in zebrafish 34. However, to the best of our knowledge, upstream modulators and signaling control systems of this pathway have not yet been discovered. The MT signaling system is a possible candidate for neuroendocrine regulation ensuring seasonal reproduction, photoperiodic breeding, and modifying skin pigmentation 37, 30, 38-40. This raises an important question: What is the role of the MT signaling system in the central neuroendocrine system and how does it control skin coloration in this marine teleost species? Herein, we addressed this question by systematic characterization of the MT signaling system in the large yellow croaker. Our results define the mitogen activated protein kinase (MAPK) cascade signaling pathways activated by different Mtnrs and reveal possible signaling patterns of MT/Mtnrs in specific central neuroendocrine cell types, focusing on a functional output of chromatophore mobilization.

## Materials and Methods

**Animals and samples collection.** The large yellow croakers (*L. crocea*) were collected from Zhoushan Fishery Research Institute. Adult female individuals (body weight: 293.42 ± 41.31 g) were used for single-cell RNA-seq and adult fish (body weight: 285.11 ± 50.43 g) were used for pharmacological experiments. The larvae and adult fish were both reared in seawater (salinity range: 30.5 to 34.0 ppt) aquaria tanks (more than 0.8 t effective water in each tank) with an indoor recirculating aquaculture system. Seawater temperature was strictly controlled at 22 °C for culturing larvae and from 22 °C to 25 °C for adult fish. The adults were anesthetized with 0.01% MS222 (E10505, Sigma-Aldrich) before sampling. All experimental procedures were carried out according to institutional regulations concerning the protection of experimental animals. The tissues were fixed with cold 4% paraformaldehyde (PFA) fix solution (Beyotime) for further histochemical analysis. Hypothalamus and pituitary samples were isolated from the whole brain of adult female large yellow croaker for single-cell sequencing.

Total RNA was extracted using TRIzol reagent (TaKaRa, Kusatsu, Japan) and phenol-chloroform (Sinopharm Chemical Reagent Co., Ltd.), following the manufacturer's instructions. High-quality total RNA (1 μg) was reverse-transcribed into single-stranded cDNA by M-MLV reverse transcriptase and oligo(dT)20 (Sangon Biotech, Shanghai, China) according to the manufacturer's instructions. The cDNA samples were stored at -80 °C until further analysis.

**Bioinformatic searches and tools.** The *mtnr* sequences from 12 vertebrate species were retrieved from GenBank (Table S1). The amino acid sequences corresponding to the Mtnr genes were translated using DNAMAN 8.0 (https://www.lynnon.com). Homology analysis of the Mtnr protein sequences was performed using the COBALT Multiple Alignment Tool (https://www.ncbi.nlm.nih.gov/tools/cobalt/cobalt.cgi?CMD=Web). The robustness phylogenetic tree was constructed using the maximum-likelihood algorithm 41 with bootstrapping 1000 times by MEGA 6.0 (http://www.megasoftware.net). NetNGlyc 1.0 Server (http://www.cbs.dtu.dk/services/NetNGlyc/), NetPhos 3.1 Server (http://www.cbs.dtu.dk/services/NetPhos/) and MEME Suite (https://meme-suite.org/meme/doc/meme.html) were used to predict N-linked glycosylation, phosphorylation sites, and motifs of *Lc*Mtnr, respectively. The *Lc*Mtnrs protein structure and function were predicted using I-TASSER (https://zhanglab.ccmb.med.umich.edu/I-TASSER/) 42.

**cDNA cloning and plasmid construction.** Primers (Table S2) were designed to clone the coding sequences of the *L. crocea mtnr* genes. The PCR products were cut with restriction enzymes (Table S2) and recombined into pEGFP-N1 (Clontech) and pCMV-FLAG (Sigma) vectors using an In-Fusion Cloning kit (TaKaRa). The constructed vectors were further sequenced to verify their orientation, sequence fidelity, and reading frame. The accession IDs of the coding sequences in NCBI are listed in Table S2.

**Cell manipulation and transfection.** All *Lc*Mtnr‐activation experiments were performed first in human embryonic kidney (HEK293) cells and were partially verified in large yellow croaker ovary fibroblast (LOF) cell lines. HEK293 and LOF cells were acquired from The National Institutes of Health (Bethesda, MD, USA) and as described previously 43, respectively. Dulbecco's modified Eagle's medium (DMEM, HyClone, USA), and DMEM/Nutrient Mixture F-12 (DMEM/F-12, HyClone, USA) supplemented with 10% heat-inactivated fetal bovine serum (FBS, Hyclone, Waltham, MA, USA) and 1% penicillin-streptomycin solution (Procell) were used to culture HEK 293 and LOF cells, respectively. HEK293 and LOF cells were grown in a humidified atmosphere with 5% CO_2_ and incubated at 37 °C and 28 °C, respectively. The vectors and plasmid constructs were transfected into cells using the FuGENE® HD transfection reagent (Promega, Cat#: E2311) according to the manufacturer's protocols.

***Lc*mtnrs localization and translocation detection by confocal microscopy.** To facilitate **observation** under a confocal microscope, HEK293 cells expressing *Lc*Mtnr1a1/1a2/1b1/1b2/1c (HEK293-LcMtnrs) and LOF cells expressing *Lc*Mtnr1c (LOF-*Lc*Mtnr1c) were seeded on coverslips overnight. To detect the receptors on the membrane surface, HEK293-*Lc*Mtnrs-EGFP and LOF-*Lc*Mtnr1c-EGFP cells were stained with the cell membrane probe DiI (Beyotime) and nuclei probe DAPI (Beyotime) following the manufacturer's instructions. For FLAG-*Lc*Mtnrs detection, HEK293-FLAG-*Lc*Mtnrs cells were labeled with the anti-FLAG M2-FITC antibody (1:100, Sigma, Cat#: F4049). For the translocation assays, HEK293-*Lc*Mtnrs-EGFP and LOF-*Lc*Mtnr1c-EGFP cells were pre-treated with or without the Mtnr1a/1b inhibitor luzindole (10 μM, Sigma) for 1 h at 37 °C and then stimulated with the indicated concentrations of melatonin (Solarbio) for an appropriate amount of time at 37 °C. After stimulation, cells were fixed and washed three times with ice-cold PBS before mounting. Images were captured using a laser scanning confocal microscope (Leica TCS SP5II) equipped with a HCX PL APO lambda blue 63×1.40 oil immersion objective.

**Calcium and cAMP measurement.** The fluorescent calcium indicator jGCaMP7 was used to image and measure changes in Ca^2+^ concentrations. HEK293 cells transiently co-transfected with pCMV-jGCaMP7 (Beyotime) and FLAG-*Lc*Mtnrs plasmid constructs were reseeded in 20 mm glass-bottom cell culture dishes overnight and then stimulated with the indicated concentrations of MT been monitored by confocal microscopy. When required, cells were pre-treated for 60 min with EGTA (5 mM, MedChemExpress) or BAPTA-AM (50 μM, MedChemExpress) prior to the initiation of the experiments. For cAMP accumulation, HEK293-FLAG-*Lc*Mtnrs cells pre-treated with phosphodiesterase inhibitor IBMX (0.01 mM, Beyotime) for 1 h were stimulated with the indicated concentrations of MT for 10 min with or without forskolin (10 μM, Beyotime) for 10 min incubation. The cAMP concentrations were assessed by a competitive binding enzyme-linked immunosorbent assay (ELISA) (Parameter cAMP assay, R&D, Minneapolis, MN) according to the manufacturer's instructions.

**Protein extraction and immunoblot analysis.** To investigate the phosphorylation of ERK, HEK293-*Lc*Mtnrs or LOF-*Lc*Mtnr1c cells incubated with different concentrations of MT. Subsequently, cells were lysed using RIPA lysis buffer (Beyotime) for 45-60 min at 4 °C. Equal amounts of total cell lysate supernatants were electrophoresed on SDS-polyacrylamide gels (PAGE) (10-12%) and transferred onto a polyvinylidene fluoride (PVDF, Millipore) membrane. Membranes were blocked in 5% bovine serum albumin (BSA, Biofroxx) in TBSTw (Beyotime) for 1 h before incubation with anti-rabbit Phospho-p44/42 ERK1/2 antibody (1:2000, Cell Signaling Technology, Cat#: 4370S) overnight. The membranes were further probed with an anti-rabbit-HRP secondary antibody (1:3000, Beyotime, Cat#: A0208).

To detect alkylamine N-acetyltransferase (AANAT) in pineal and intestinal tissues, tachykinin precursor 1 (TAC1, cleavage to tachykinins substance P (SP) and neurokinin A (NKA)) and melanin-concentrating hormone (MCH) in the hypothalamus, and α-melanocyte-stimulating hormone (α-MSH) in the pituitary of the large yellow croaker, all tissues were sampled and homogenized in RIPA buffer for 45 min at 4 °C. Equal amounts of protein (20 μg) from each sample were electrophoresed on 15% SDS-PAGE and transferred onto PVDF membranes (230 mA, 30 min for TAC1 and MCH detection, and 60 min for AANAT, α-MSH, and protein control detection), which were analyzed by immunoblotting with indicated antibodies (Table S3).

Immunoreactive bands were detected using an enhanced chemiluminescence substrate (1:3000, Beyotime, Cat#: A0208) and captured using a Tanon 5200 chemiluminescent imaging system (Tanon Science & Technology, Shanghai, China). The intensities of the immunoblots were visualized and quantified using a Bio-Rad Quantity One imaging system (Bio-Rad Laboratories).

**Real-time quantitative PCR (qRT-PCR).** The qRT-PCR primers (Table S2) for both the control and the *Lcmtnrs* gene coding sequence regions were designed using Primer Premier 5 (Premier) and pre-tested to ensure amplification of single discrete bands. *Lcmtnrs* transcript levels were determined using the SYBR PrimeScript™ RT reagent Kit (TaKaRa), following the manufacturer's instructions. The relative expression of *Lcmtnrs* was determined using the ABI 7500 Software v2.0.6 (Applied Biosystems) using the 2^-ΔΔCt^ algorithm. *β-Actin* was selected as a reference gene and used for data normalization 44-46.

***In situ* Hybridization Chain Reaction (HCR) and immunofluorescence assays.** The paraffin-embedded tissue sections were deparaffinized and rehydrated for next steps. For *in situ* HCR assay, pre-hybridization was performed in the hybridization buffer for 30 min inside a 37 °C humidified chamber, and then the slides were incubated with probe solution overnight at 37 °C. After washing in a series of wash solutions, the slides were incubated with pre-amplification buffer for 30 min at 25 °C and fluorescently labeled hairpins were snap-cooled before use. After overnight (12-16 h) incubation at 25 °C with the hairpin solution (lot number list in Table S3) in the dark, the sections were washed and mounted for imaging. Hybridization buffer (LOT.BPH02822), HCR probes, wash solution (LOT.BPW30122), amplification buffer, and fluorescently labeled hairpins were purchased commercially (Molecular Instruments Inc., CA, USA). The HCR probes were targeted to specific genes based on their RefSeq ID (Table S3).

For immunofluorescence assays, all prepared sections were incubated with an antigen retrieval buffer (Beyotime) at 96 °C for 10 min. After cooling, the sections were blocked with 5% goat serum (Beyotime) in PBS containing 0.3% Triton X-100 (Beyotime) and incubated with primary antibodies (Table S3) overnight at 4 °C. The corresponding secondary antibodies (FITC-labeled and Cy3 labeled, 1:500, all purchased from Beyotime) were incubated with the slides at room temperature for 2 h. After washing three times in PBS, the slides were stained with DAPI to mark the nuclei.

Using an anti-fade mounting medium (Solarbio) to seal and mount the sections, all images were captured using a confocal microscopy (FV3000) system (2.3.1.163 version). For immunofluorescence detection, excitation at 405 nm was filtered for emission in the 430 - 470 nm range, while 514 nm was filtered for the 530 - 630 nm range. For HCR detection, excitation was performed at 405, 488, 561, and 640 nm, and fluorescence detection was performed using 520 ± 20, 580 ± 50, 595 ± 25, and 700 ± 50 nm bandpass filters.

**Single-cell dissociation, libraries construction, and sequencing.** For single-cell dissociation, hypothalamic and pituitary tissues isolated from the whole brain were transferred to sterile-filtered PBS and then digested by incubation with Accutase (Sigma) for 20 min at 24 °C. Subsequently, the mixture was filtered and washed with ice-cold PBS to arrest digestion. The cells were then concentrated by centrifugation at 2500 rpm for 5 min at 4 °C. The concentrated cells were carefully resuspended in ice-cold sterile PBS and transferred to tubes. The cells (viability exceeding 80%) were adjusted to a final concentration of 1,000 cells µL^-1^ for further processing.

All single-cell suspensions were loaded into a chromium controller (10X Genomics, Pleasanton, CA, USA) to generate single-cell gel beads-in-emulsions. Single-cell cDNA libraries were prepared, and sequencing was performed by BGI using the 10x Genomics Platform (Shenzhen, China). Cell Ranger (https://support.10Xgenomics.com/single-cell-gene-expression/software/downloads/) was used to perform sample demultiplexing, read alignment, feature-barcode matrix generation, and single-cell gene unique molecular index (UMI) counting. The genomic index was made in Cell Ranger (10X genomic) using the *L. crocea* genome version 2.0 (accession number: GCA_000972845.2). Cell Ranger output matrices for the hypothalamus and pituitary with 5-10 biological and two technical replicates were used for further analysis in the Seurat v4.0 R package 47.

**Data processing and analysis.** To control the quality of single-cell RNA-seq data, genes expressed in fewer than three cells and cells expressing fewer than 200 genes were excluded. Cells whose percentage of expressed mitochondrial genes was greater than 20% or those whose unique molecular identifier (UMI) counts were either less than or greater than one IQR distance outside the UMI counts were also excluded. Subsequently, the raw counts matrix was normalized and centered with the 'LogNormalize' normalization method (scale factor set at 10,000) and the ScaleData command. Multiple biological replicate data were integrated using canonical correlation analysis (CCA) implemented in Seurat's RunMultiCCA function.

The hypothalamic and pituitary sample datasets were aligned and clustered independently. First, genes with high variance were determined and implemented using FindVariableGenes with default parameters for further principal component analysis (PCA). Next, the top 50 principal components were clustered using a shared nearest neighbor (SNN) modularity based on the Jack Straw plot, as implemented in FindClusters with 50 dimensions (resolution augmentation set to 3.0). Finally, to perform clustering dimensionality reduction, Uniform Manifold Approximate and Projection (UMAP) was used and implemented on the first 50 principal components. The differential expression for clusters was implemented using FindMarkers. Cluster identities were determined using the established marker genes (http://zfin.org/) and databases (http://biocc.hrbmu.edu.cn/CellMarker/).

**Pharmacological experiments.** Pharmacological experiments were performed on *L. crocea* adults and larvae. Adult fish were injected intraperitoneally with 0.3 mL saline solution (0.9% NaCl) containing the indicated concentration of MT or 0.9% saline (control). The larvae were kept in 10 cm cell culture dishes (Corning) during the experiments and pre-incubated with the indicated Mtnr antagonist for 2 h to attenuate or block the effects of MT administration.

**Chroma measurement and pigment granule mobilization analysis.** After chemical treatments, color quantification of the ventral and dorsal skins was performed on a spectrophotometer (DS-700D, Caipu, China) with a measurement area of 8 mm caliber. In accordance with the criteria of the Commission Internationale de l'Eclairage, we used the color parameters L*, a*, and b* as lightness, red/green intensity, and yellow/blue intensity, respectively.

Fish scales from the ventral and dorsal skins were isolated and fixed with 4% PFA overnight at 4 °C, and then images of xanthophores or melanophores were acquired using a stereomicroscope. For adult fish, the chromatophore-covered area was selected using an oval tool and measured using ImageJ. For larvae, the width of a chromatophore, defined as the distance between the ventral midpoint extending to the back edge of the chromatophore, was scaled using the scale tool in Adobe Suite Photoshop CS6.

**ELISA for neurohormone concentration analysis.** After chemical administration, the whole brain and blood from the caudal tail vein of anesthetized adult fish, as well as whole larvae, were collected for MT, corticotropin-releasing hormone (CRH), MSH, and MCH detection. The blood samples were pre-centrifuged (1000 × g, 15 min) at 4 °C, and the plasma was stored at -80 °C. All samples were analyzed by ELISA (Jianglai Biotech, China) following the manufacturer's instructions.

**Statistical analysis.** All data are presented as mean ± standard error of the mean (SEM), and statistical significance was assessed using one-way analysis of variance (ANOVA) followed by Tukey's multiple comparisons test using GraphPad Prism software (version 6.0). Statistical significance was set at *P* < 0.05. All the experimental data were obtained from at least three independent experiments.

## Results

**Figure 1:**
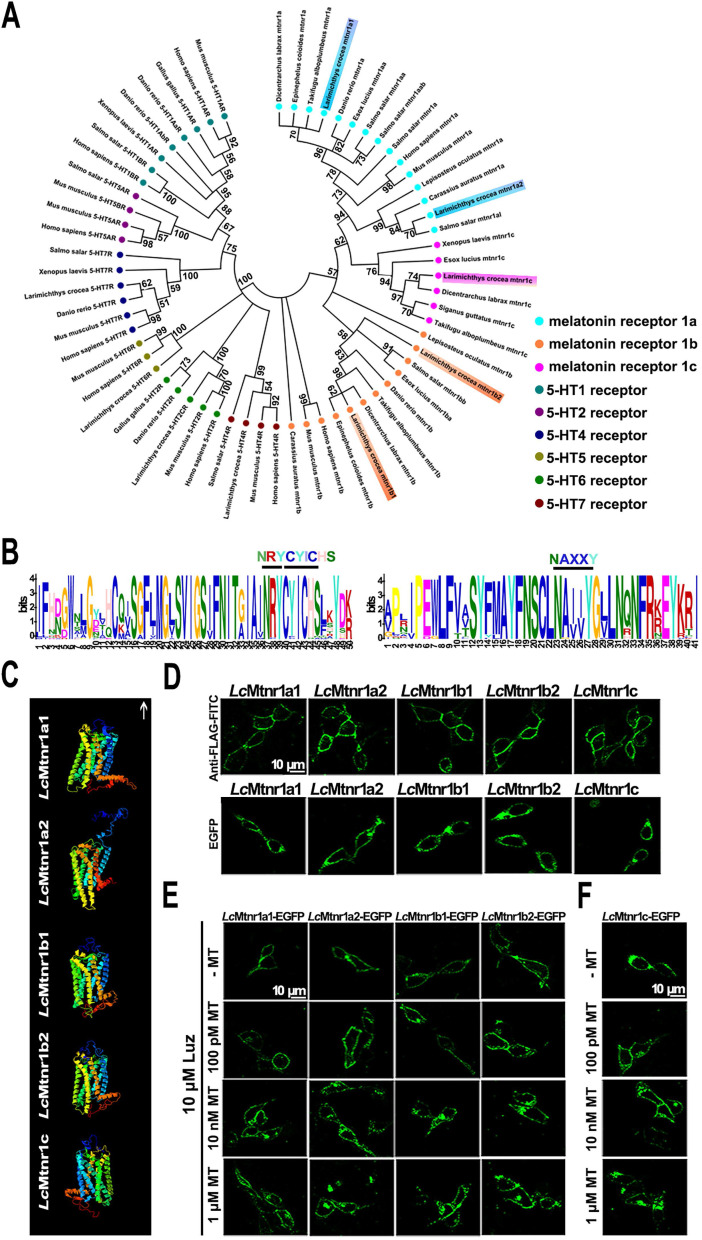
** Molecular characterization of *Larimichthys crocea* melatonin receptors.** *(A)* Phylogenetic tree of the melatonin receptors with a 5-hydroxytryptamine (5-HT) receptors out-group. The tree was constructed by the Maximum Likelihood method using amino acid sequences of melatonin receptors (Mtnrs) and 5-HT receptors (5-HTRs). Bootstrap values > 50% are indicated above branches. Colors indicate individual groups. Sequence IDs are listed in Table S1. *(B)* Conservative motif prediction of the *L. crocea* melatonin receptors (*Lc*Mtnrs). The length of the amino acid is inferred by the ruler at the bottom. Different colors of letters represent different kinds of amino acids residues, and the size of letters represents the frequency of amino acid occurrence. Conserved amino acid residues of the *Lc*Mtnrs are marked above. *(C)* The three-dimensional structure of the *Lc*Mtnrs was predicted via I-TASSER. The transmembrane domains are in seven different colors; the upward arrow indicates extracellular direction. *(D)* Subcellular location of *Lc*Mtnrs in HEK293 cells. “Anti-FLAG-FITC” refers to stable FLAG-*Lc*Mtnrs-expressing HEK293 cells that were labeled with the anti-FLAG-FITC (M2) antibody. “EGFP” refers to stable *Lc*Mtnrs-EGFP-expressing HEK293 cells without MT stimulation. *(E) Lc*Mtnrs-EGFP internalization induced by MT in HEK293 cells. The cells stably expressing *Lc*Mtnr1a1/1a2/1b1/1b2-EGFP were pre-treated with 10 μM luzindole (Luz) at 37 °C for 60 min and then stimulated with 100 pM, 10 nM and 1 μM MT for an additional 30 min. *(F) Lc*Mtnr1c-EGFP internalization induced by MT in HEK293 cells. The cells stably expressing *Lc*Mtnr1c-EGFP were stimulated with 100 pM, 10 nM and 1 μM MT for 30 min. The results are representative of three independent experiments. MT: melatonin; 5-HT: 5-hydroxytryptamine (serotonin); Luz: Luzindole.

**MAPK activation mediated by *Lc*Mtnrs via G_αi_, G_αq_, or G_αi_+G_αs_ protein-coupled signal transduction pathways.** Five *Lc*Mtnrs were defined as *Lc*Mtnr1a1/2, *Lc*Mtnr1b1/2 and *Lc*Mtnr1c by systematic *in silico* analyses (Fig. 1A-B, and Fig. S1-8, GenBank accession IDs listed in Table S1).

**Figure 2:**
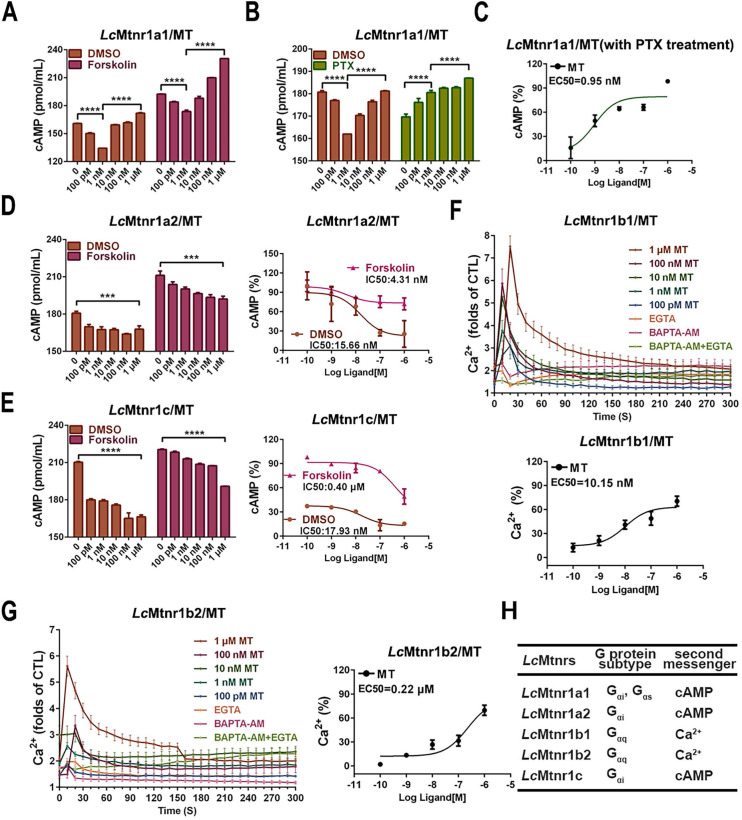
**Cell signaling characteristics of *Larimichthys crocea* melatonin receptors.** *(A-B)* The cAMP accumulation in *Lc*Mtnr1a1-expressing HEK293 cells. HEK293-*Lc*Mtnr1a1 cells were incubated with 10 μM forskolin for 15 min *(A)* or PTX for 16 h *(B)* and then stimulated with indicated concentrations of MT for an additional 15 min. *(C)* The inhibition efficiency in cAMP accumulation in *Lc*Mtnr1a1-expressing cells in the presence of PTX. *(D-E)* The dose-dependent inhibition of forskolin-induced cAMP accumulation in *Lc*Mtnr1a2-expressing and *Lc*Mtnr1c-expressing HEK293 cells. HEK293-*Lc*Mtnr1a2 cells *(D)* and HEK293-*Lc*Mtnr1c cells *(E)* were incubated with 10 μM forskolin for 15 min and then stimulated with indicated concentrations of MT for an additional 15 min. Ca^2+^ mobilization in FLAG-*Lc*Mtnr1b1-expressing *(F)* and FLAG-*Lc*Mtnr1b2-expressing *(G)* HEK293 cells were measured in response to indicated concentrations of MT with pre-treated intracellular calcium chelator (BAPTA-AM, 50 μM), extracellular calcium chelator (EGTA, 5 mM), or BAPTA-AM+EGTA, respectively. *(H)* The receptor coupled G-protein subtypes and intracellular second messenger induced by* Lc*Mtnrs. Data are presented as means ± SEM of three independent experiments. Data are analyzed using one-way ANOVA with Tukey's multiple comparison test, *** P < 0.001, **** P < 0.0001. MT: melatonin; PTX: pertussis toxin; CTL: control; EGTA: an extracellular calcium chelator; BAPTA-AM: an intracellular calcium chelator.

The subcellular localization and ligand-activated internalization of *Lc*Mtnrs were evaluated by confocal microscopy using FLAG-*Lc*Mtnrs or *Lc*Mtnrs-EGFP recombinant expressing HEK293 or large yellow croaker ovary fibroblast (LOF) cell lines (Fig. 1C-F and S9). The intracellular second messengers (cAMP and Ca^2+^) were assessed in HEK293-FLAG-*Lc*Mtnrs cells treated with various concentrations of MT with or without pre-incubation with 5 mM extracellular calcium chelator (EGTA), 50 μM intracellular calcium chelator (BAPTA-AM), or 50 ng/mL pertussis toxin (PTX). *Lc*Mtnr1a1 was activated evidenced by increased intracellular cAMP levels (from 1 nM to 1 μM MT) following a downward trend (< 1 nM MT) (Fig. 2A-C). In contrast, intracellular cAMP accumulated in a ligand dose-dependent manner after pre-treatment with PTX, which catalyzes ADP-ribosylation of G_i/o_ thereby blocking receptor coupling. Both *Lc*Mtnr1a2 and *Lc*Mtnr1c were activated by MT, inducing a significant decrease in intracellular cAMP levels (Fig. 2D-E). Although with no significant ligand induced cAMP alteration in *Lc*Mtnr1b1/2 expressing cells, MT-elicited Ca^2+^ mobilization was detected (Fig. S10A and Fig. 2F-G) and fully blocked by pre-treatment with EGTA and BAPTA-AM (Fig. 2F-G). However, there were no significant calcium signal changes detectable in MT-treated *Lc*Mtnr1a1/1a2/1c-expressing cells (Fig. S10B). The receptor coupled G-protein subtypes and second messenger induced by* Lc*Mtnrs were summarized in Fig. 2H.

Furthermore, we examined MT-mediated activation of ERK1/2 in *Lc*Mtnrs-expressing cells by ERK1/2 phosphorylation. Fig. S11 shows the activation of ERK1/2 phosphorylation mediated by all five tested *Lc*Mtnrs, while in the HEK293 cells, neither the blank nor pFLAG-CMV-3 vector-expressing cells, showed any significant MT response. Moreover, upon MT stimulation, activation of ERK1/2 phosphorylation mediated by *Lc*Mtnrs was detected in a dose (5 min) and time course (100 nM MT) dependent manner (Fig. S12). As shown in Fig. S13, MT-induced ERK1/2 activation peaked within 10 min. And this phosphorylation was significantly inhibited by pretreatment with 10 μM luzindole (*mtnr1a/1b* antagonist) or 0.1 μM prazosin hydrochloride (*mtnr1c* antagonist) (Fig. S14).

**Figure 3:**
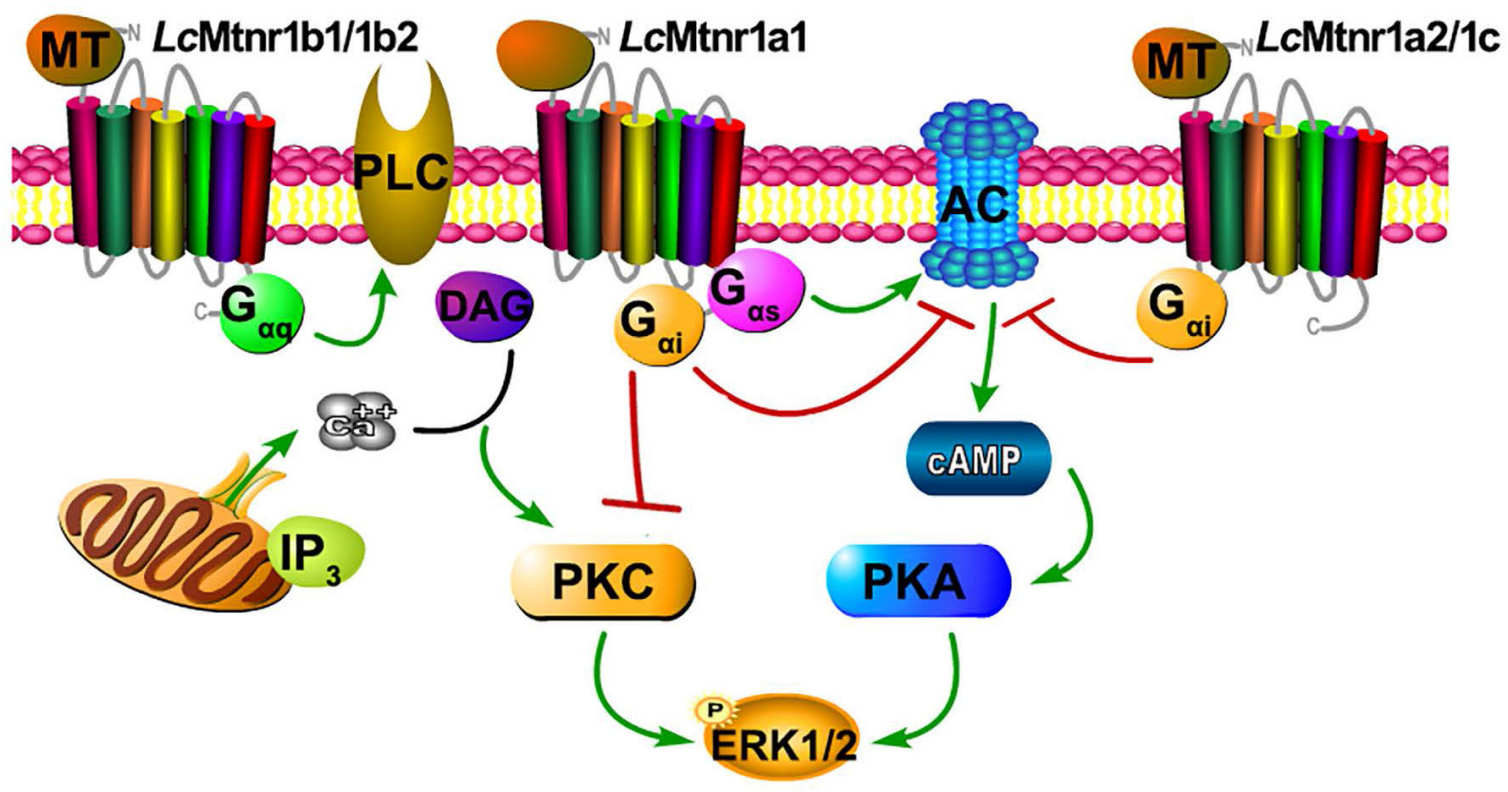
** Schematic diagram of *Larimichthys crocea* melatonin receptor activation.** The agonist MT binding to *Lc*Mtnr1a1 activates both G_αi_ and G_αs_ families of heterotrimeric G proteins, which leads to dissociation of the G protein subunits G_βγ_, enhances PKA activity, inhibits PKC activity, and stimulates phosphorylation of ERK1/2. The agonist MT binding to *Lc*Mtnr1a2 or *Lc*Mtnr1c activates G_αi_ families of heterotrimeric G proteins, which leads to dissociation of the G protein subunits G_βγ_, inhibits adenylate cyclase activity leading to intracellular cAMP accumulation reduction, and stimulates phosphorylation of ERK1/2. The agonist MT binding to *Lc*Mtnr1b1 or *Lc*Mtnr1b2 activates G_αq_ families of heterotrimeric G proteins, which leads to dissociation of the G protein subunits G_βγ_, and activates PLC, leading to intracellular Ca^2+^ mobilization and ERK1/2 phosphorylation. The diagram was generated by Pathway Builder Tool 2.0. All pictures and data shown are representative of at least three independent experiments. PLC: Phospholipase C; AC: Adenylate Cyclase; cAMP: cyclic adenosine monophosphate; PKA: Protein Kinase A; ERK1/2: Extracellular signal-Regulated Kinase1/2; PKC: Protein Kinase C; DAG: Diacylglycerol; IP_3_: Inositol triphosphate; P: phosphorylation.

To verifying the G protein subtypecoupling and following cell signaling, we pre-treated *Lc* Mtnrs expressing cells with PTX, FR-900359 (a specific inhibitor of G_αq_), PKA inhibitor H89, and PKC inhibitor Go 6983. The *Lc*Mtnr1a1 response was partially inhibited by either H89 or Go 6983, while the *Lc*Mtnr1a2 and *Lc*Mtnr1c responses were both fully arrested by PTX (Fig. S15A-C). Notably, both* Lc*Mtnr1b1 and *Lc*Mtnr1b2 responses were significantly blocked by FR-900359 (Fig. S15D-E and S10A). These data reveal that ERK1/2 activation is G_αi_-dependent for *Lc*Mtnr1a2 and *Lc*Mtnr1c, G_αi_ and G_αs_ dual-dependent for *Lc*Mtnr1a1, and G_αq_-dependent for *Lc*Mtnr1b1 and *Lc*Mtnr1b2 (Fig. 3).

**Figure 4:**
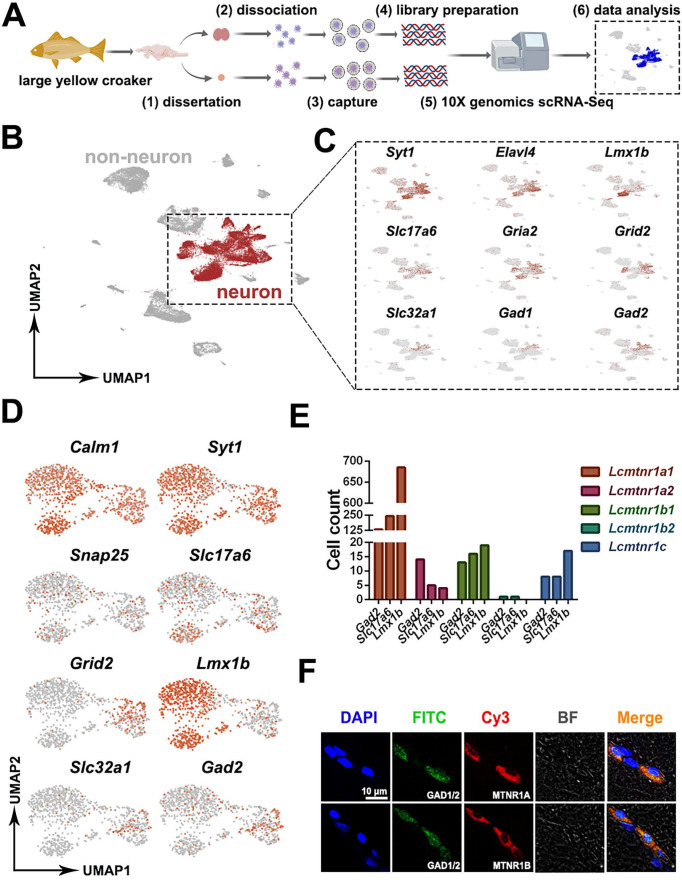
**Overall experimental design and characterization of neuronal cells among the hypothalamus cells.** *(A)* Experimental design of 10X sc-RNA-seq. Hypothalamus and pituitary cells were collected from adult female large yellow croaker and then processed via sc-RNA-seq using the 10x-Based Genomics platform. *(B-D)* UMAP plots showing cell types of the hypothalamus colored by their associated cluster identity, expression of neuron markers *synaptotagmin 1*(*Syt1), ELAV like neuron-specific RNA binding protein 4 (Elavl4), LIM homeobox transcription factor 1 beta (Lmx1b), solute carrier family 17 member 6 (Slc17a6), glutamate ionotropic receptor AMPA type subunit 2 (Gria2), glutamate ionotropic receptor delta type subunit 2 (Grid2), solute carrier family 32 member 1 (Slc32a1), glutamate decarboxylase 1 (Gad1) and glutamate decarboxylase 2* (*Gad2*), co-expression of *Lcmtnrs* and neuron marker genes. *Snap25*: *synaptosome associated protein 25*; *Syt1*: *synaptotagmin 1*;* Calm1*: *Calmodulin 1*; *Lmx1b*: *LIM homeobox transcription factor 1 beta*; *Slc17a6*: *solute carrier family 17 member 6*; *Gria2*: *glutamate ionotropic receptor AMPA type subunit 2*; *Grid2*: *glutamate ionotropic receptor delta type subunit 2*; *Slc32a1*: *solute carrier family 32 member 1*; *Gad1*: *glutamate decarboxylase 1*; *Gad2*: *glutamate decarboxylase 2*;* (E)* Histogram showing the counts of the cells co-expressing *Lcmtnrs* and neuron marker genes (*Gad2*, *Slc17a6* and *Lmx1b*). *(F)* Double immunofluorescence of Gad1/2 and Mtnr1a/1b. Representative photomicrographs of sections through a hypothalamic section showing the same area immunolabelled for Gad1/2 (green), and for Mtnr1a/1b (red), with merged images in (yellow). Scale, as indicated. BF: bright field.

**Patterns of MT signaling in the neuroendocrine system.** We isolated hypothalamic and pituitary tissues from adult fish and dissociated them for scRNA sequencing (Fig. 4A). 9,900-12,099 cells per sample were recovered, with 64,708-79,072 reads per cell and 19,279-20,188 genes detected (BioProject ID on NCBI: PRJNA814756). After eliminating redundancies in the raw data, scRNA data from 25,816 healthy hypothalamus cells (5 individuals, 2 replicates) and 17,065 healthy pituitary cells (10 individuals, 2 replicates) were collected for downstream analyses.

To identify neuronal cell types in the hypothalamus, a set of highly variable genes were screened and used for UMAP dimensionality reduction analysis. We characterized neuronal and non-neuronal cell clusters based on the expression of pan-neuronal markers 48 (*Snap25* and *Syt1*, respectively) (Fig. 4B). 11,463 neuronal cells highly expressed *synaptotagmin 1* (*Syt1*), *ELAV like neuron-specific RNA binding protein 4* (*Elavl4*), *LIM homeobox transcription factor 1, beta b* (*Lmx1b*), *solute carrier family 17 member 6* (*Slc17a6*), *glutamate ionotropic receptor AMPA type subunit 2* (*Gria2*), *glutamate ionotropic receptor delta type subunit 2* (*Grid2*), *solute carrier family 32 member 1* (*Slc32a1*), *glutamate decarboxylase 1* (*Gad1*) and *glutamate decarboxylase 2* (*Gad2*) were shown in UMAP plots (Fig. 4C). To determine whether the MT signaling system is involved in the regulation of neurophysiological activities, the co-expression of neuronal marker genes and the *Lcmtnrs* genes were further investigated. As shown in Fig. 4D, the glutamatergic neuron marker genes *Slc17a6* and *Grid2*, dopaminergic neuron marker gene *Lmx1b*, and GABAergic neuron marker genes *Slc32a1* and *Gad2* were all retrieved in our datasets, with a differential gene expression profile. Results indicated that the *Lcmtnr1a1*-expressing neurons were indicated as the largest group in *Lc*Mntnrs gene-expressing hypothalamic neurons (Fig. 4E). The immunofluorescence detection in the hypothalamus using antibodies against vertebrate MTNR1A, MTNR1B, and GAD1/2 was further performed to verify the predicted co-expression relationship of *Lcmtnrs* and *Gad1/2*. We found that GAD1/2^+^ cells co-localized with MTNR1A or MTNR1B (shown in Fig. 4F; the antibody specificities were pre-evaluated as shown in Fig. S16).

To further identify the pattern of the MT signaling system and its downstream hormonal networks, we investigated the existence of well-documented neuropeptides in neuronal cells co-expressed with *Lcmtnrs* genes. Most of the neuropeptide genes were detected in *Lcmtnr1a1*-expressing cells, while all five *Lcmtnrs* genes were successfully detected in* Acyl-CoA binding domain containing 7 (Acbd7)*-, *adenylate cyclase activating polypeptide 1 (Adcyap1)*-, *apelin (Apln)*-, *cerebellin 2 precursor (Cbln2)*-,* tachykinin precursor 1 (Tac1)*-,* secretogranin II (Scg2)*-,* secretogranin V (Scg5)*- and* VGF nerve growth factor inducible (Vgf)-* expressing hypothalamus neurons (Fig. S17). Neuropeptide receptors in hypothalamus neurons (Fig. S18A) or pituitary cells (Fig. S18B) were also analyzed based on ligand-receptor interaction pairs.

**Figure 5:**
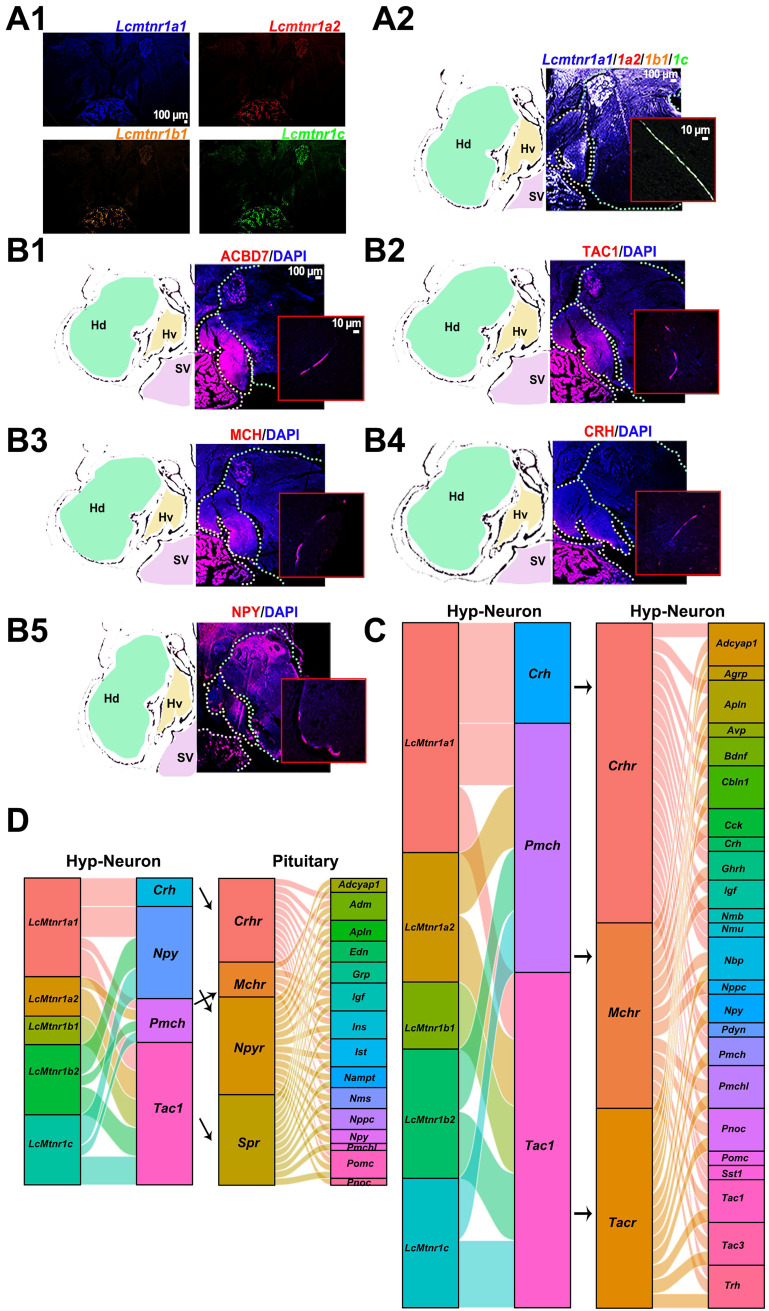
**The interaction between *Larimichthys crocea* melatonin receptors, neuropeptides and neuropeptide receptors.** *(A)* Distribution of *Lc*Mtnr1a1/1a2/1b1/1c mRNA analyzed by HCR assays. Representative photomicrographs of sections through a hypothalamus showing *Lcmtnr1a1*, *Lcmtnr1a2*,* Lcmtnr1b1* and *Lcmtnr1c* mRNA HCR are in blue, red, orange and green, respectively *(A1)* or merged *(A2)*. Red boxes indicate multiple successive positive fibers in the hypothalamus. Hv: the ventral zones of the hypothalamus; Hd: the dorsal zones of the hypothalamus; Sv: Saccus vasulosus. *(B)* Distribution of neuropeptides (ACBD7, TAC1, MCH, CRH and NPY) analyzed by immunofluorescence technic. Representative photomicrographs of sections through a hypothalamus showing the same area immunolabelled for ACBD7, TAC1, MCH, CRH and NPY (red), and for nuclei (blue). *(C-D)* Co-expression of *Lc*Mtnrs and neuropeptides in neuronal cells, and the interaction between the *Lc*Mtnrs and neuropeptides and their receptors from hypothalamus neurons *(C)* and pituitary cells *(D)*. The diagram was generated by the Xiantao web (https://www.xiantao.love). ACBD7: Acyl-CoA binding domain containing 7; TAC1: Tachykinin precursor 1; MCH: Melanin-concentrating hormone; CRH: Corticotropin-releasing hormone; NPY: neuropeptide Y.* Crhr: corticotropin-releasing hormone receptor; Mchr: melanin concentrating hormone receptor; Mcr: mast cell regulator; Agrp: agouti related neuropeptide; Apln: apelin; Avp: arginine vasopressin; Bdnf: brain derived neurotrophic factor; Cbln1: cerebellin 1; Cck: cholecystokinin; Crh: corticotropin releasing hormone; Gnrh: growth hormone releasing hormone; Igf: insulin-like growth factor; Nmb: neuromedin B; Nmu: neuromedin U; Npb: neuropeptide B; Nppc: natriuretic peptide C; Npy: neuropeptide Y; Pdyn: prodynorphin; Pmch: pro-melanin concentrating hormone; Pmchl: pro-melanin-concentrating hormone, like; Pnoc: prepronociceptin; Pomc: proopiomelanocortin; Sst1: susceptibility to tuberculosis 1; Tac1: tachykinin precursor 1; Tac3: tachykinin precursor 3; Trh: thyrotropin releasing hormone; Spr: sepiapterin reductase; Adm: adrenomedullin; Grp: gastrin releasing peptide; Npyr: neuropeptide Y receptor; ins:* insulin;* ist: IST1 factor associated with ESCRT-III; Nampt: nicotinamide phosphoribosyltransferase; nms: neuromedin S.*

We next examined the distribution of *Lcmtnrs* and related neuropeptides in the hypothalamus using HCR and immunohistochemistry assays. *Lcmtnr1a1*^+^ neurons were widely distributed in the hypothalamus (Fig. 5A1-2; hypothalamus structure shown in Fig. S19) and *Lcmtnr1a2*^+^, *Lcmtnr1b1*^+^ and *Lcmtnr1c*^+^ neurons were few in the ventral (Hv) and dorsal (Hd) zones of the hypothalamus. Interestingly, multiple successive positive fibers were observed in most HCR staining sections, suggesting co-expression of different Mtnr types in single neurons with combined biological functions (Fig. 5A2). ACBD7^+^, TAC1^+^ and MCH^+^ neurons were mainly distributed in the Hv area (Fig. 5B1-3), while immunoreactivity for neuropeptide Y (NPY) protein was detectable in both the Hv and Hd areas of the hypothalamus (Fig. 5B5). However, few CRH^+^ cells expressed in Hv were detected (Fig. 5B4).

**Figure 6:**
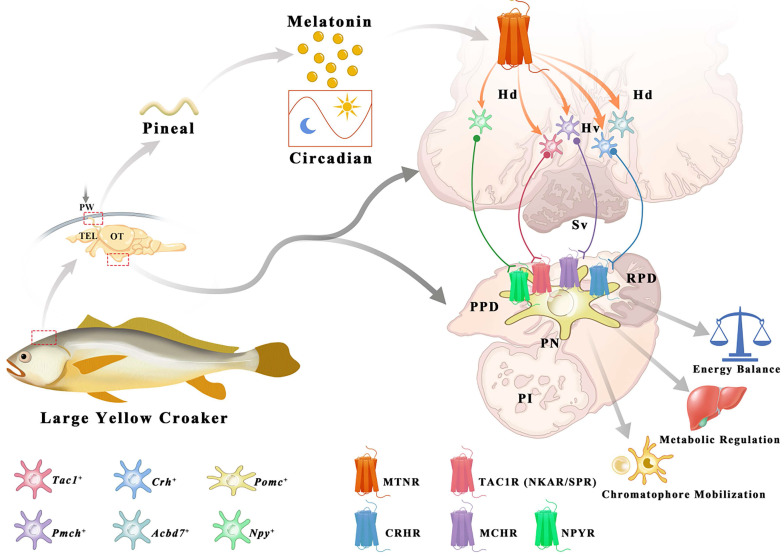
** Schematic diagram illustrating the potential role of melatonin signaling system in orchestrating neuroendocrine functions in the large yellow croaker.** Melatonin is secreted during the nighttime by the pineal gland. Once stimulated by MT, *Lc*Mtnrs are activated, leading to the expression and release of downstream neuropeptides in the hypothalamus (including the TAC1, CRH, MCH, ACBD7 and NPY). These neuropeptides promote its receptors to regulate the functions of pituitary *Pomc*^+^ neurons, ultimately carrying out corresponding biological functions such as metabolism, energy balance, and chromatophore mobilization in the large yellow croaker. Hv: the ventral zones of the hypothalamus; Hd: the dorsal zones of the hypothalamus; RPD: rostral pars distalis; PPD: proximal pars distalis; PI: pars intermedia; PN: neurohypophysis. MT: Melatonin; ACBD7: Acyl-CoA binding domain containing 7; TAC1: Tachykinin precursor 1; MCH: Melanin-concentrating hormone; CRH: Corticotropin-releasing hormone; NPY: neuropeptide Y.

The interaction profiles of MCH, TAC1, CRH and NPY, and downstream signaling factors have been constructed from scRNA-seq data. Three MT regulated peptidergic neurons, *Crh*^+^,* pro-melanin-concentrating hormone (Pmch)*^+^ and *Tac1*^+^ neurons, were found to have a strong association with a group of hypothalamic neurons including the *Pmch^+^* and *pro-melanin concentrating hormone, like (Pmchl)^+^* neurons (Fig. 5C). The pituitary *adrenomedullin* (*Adm*)*^+^, insulin* (*Ins*)*^+^, insulin-like growth factor* (*Igf*)*^+^, IST1 factor associated with ESCRT-III* (*Ist*)*^+^, and proopiomelanocortin* (*Pomc*)*^+^* neurons were mostly regulated by neuropeptide hormones (CRH, NPY, MCH or mature peptide encoded by *Tac1* (Substance P, SP) (Fig. 5D). Collectively, scRNA-seq and the spatial distribution analyses of *Lc*Mtnrs and related neuropeptides uncover a relationship between the MT signaling system and downstream neurohormones (Fig. 6, and Fig. S20). One simplified pathway can be proposed: once stimulated by MT, following circadian rhythm, *Lc*Mtnrs are activated, leading to the release of downstream neuropeptides in the hypothalamus (including TAC1 and MCH), which promote their receptors to regulate the functions of pituitary neurons (including the* Pomc^+^* neurons), ultimately affecting physiological activities such as metabolism, energy balance, and pigment changes in the large yellow croaker.

**Figure 7:**
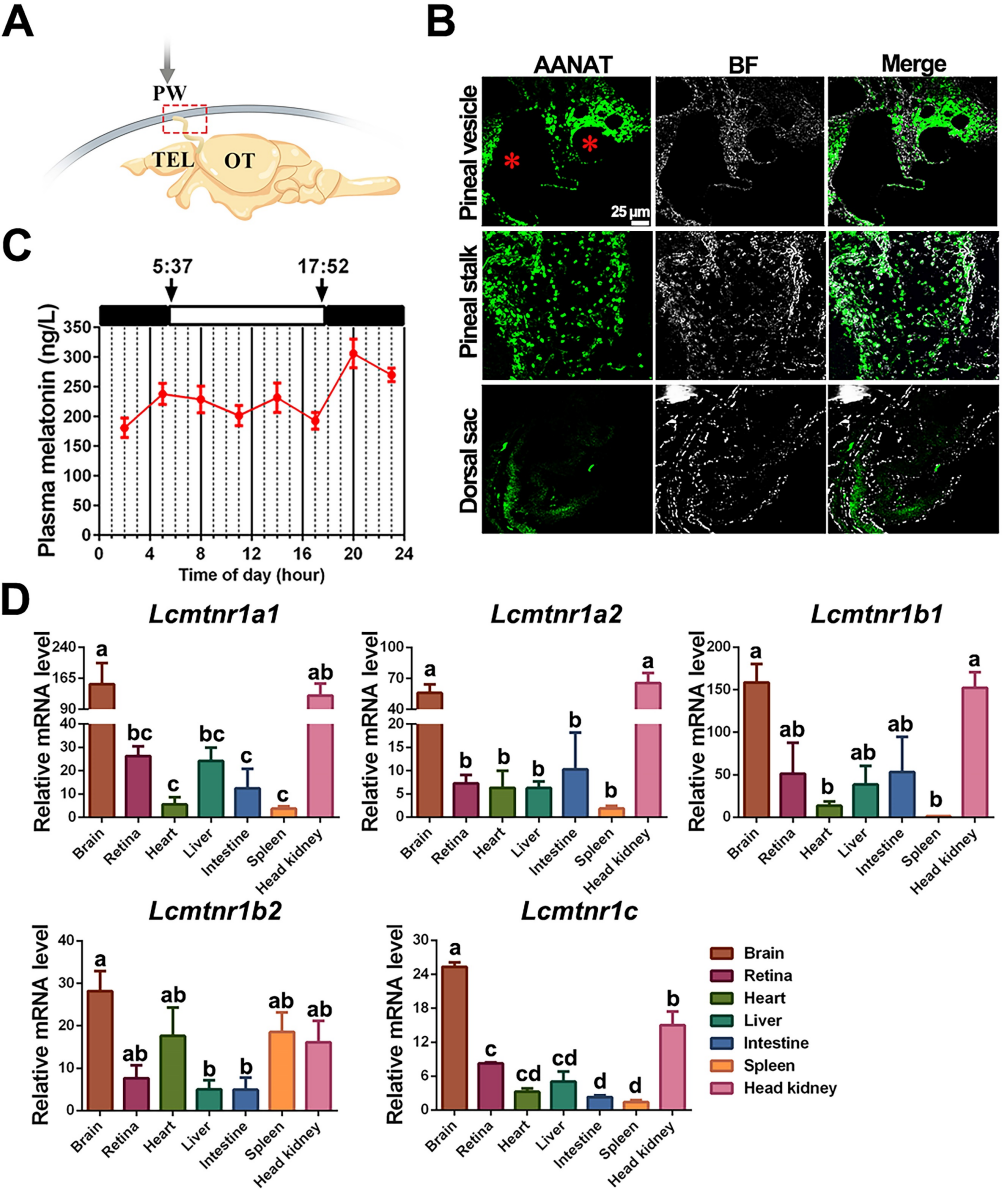
**Physiological characteristics of melatonin and melatonin receptors in the large yellow croaker.** *(A)* Schematic diagram of the location of the pineal gland in the large yellow croaker brain. The pineal gland is marked by a red box. PW, pineal window; TEL, telencephalon; OT, optic tectum. *(B)* Immunohistochemical identification of presumed MT-synthesizing cells in the pineal organ of the large yellow croaker. “AANAT” refers to the pineal sections that were incubated with the anti-AANAT antibody. BF, Bright Field. “*” refers to the pineal lumen. *(C)* Circadian variations of plasma melatonin concentration in the female large yellow croaker in autumn. Samplings were performed every three hours (02.00, 05.00, 08.00, 11.00, 14.00, 17.00, 20.00, 23.00). White and black bars represent times of light and darkness, respectively. 5:37 and 17:52 were dawn and dark, respectively. *(D)* The relative expression of *Larimichthys crocea* melatonin receptors (*Lc*Mtnrs) mRNA in the brain, retina, heart, liver, intestine, spleen, and head kidney of the large yellow croaker. Expression values were normalized against the expression of the internal control gene (*β-actin*). Each symbol and the vertical bar represents mean ± SEM (n=3). Data were analyzed using one-way ANOVA followed by Tukey's multiple comparison test, and different lowercase letters above the bars indicate significant differences (*P* < 0.05) between different tissues. AANAT: Alkylamine N-acetyltransferase.

**Secretion and distribution of MT and its receptors in adult large yellow croaker.** The pineal gland of the large yellow croaker is in a translucent pineal window below the skull, consisting of the pineal vesicle, pineal stalk, and dorsal sac (Fig. 7A). Both pineal vesicles and stalks were observed with abundant AANAT (alkylamine N-acetyltransferase)-immunopositive cells (Fig. 7B). Besides, immunoreactivity was also detected in the intestine (Fig. S21). The plasma MT demonstrated a day-night variation with daytime levels lower than those at nighttime, with the peak value detected at 20:00 (305.904 ng/L) while the mean value of MT content in the daytime (four sampling times) was 213.414 ng/L (Fig. 7C). Gene expression analysis revealed that all five *Lc*Mtnrs were found in all measured tissues with a broad distribution pattern, but comparatively high mRNA levels were detected in the brain and head kidney (Fig. 7D). Specifically, the two *mtnr1a*-paralogs shared similar expression patterns, whereas the two Mtnr1b-paralogs, *Lcmtnr1b1* and *Lcmtnr1b2*, showed different distribution profiles: the relative mRNA expression levels of *Lcmtnr1b1* in the heart and spleen were much lower than those in* Lcmtnr1b2*.

**Figure 8:**
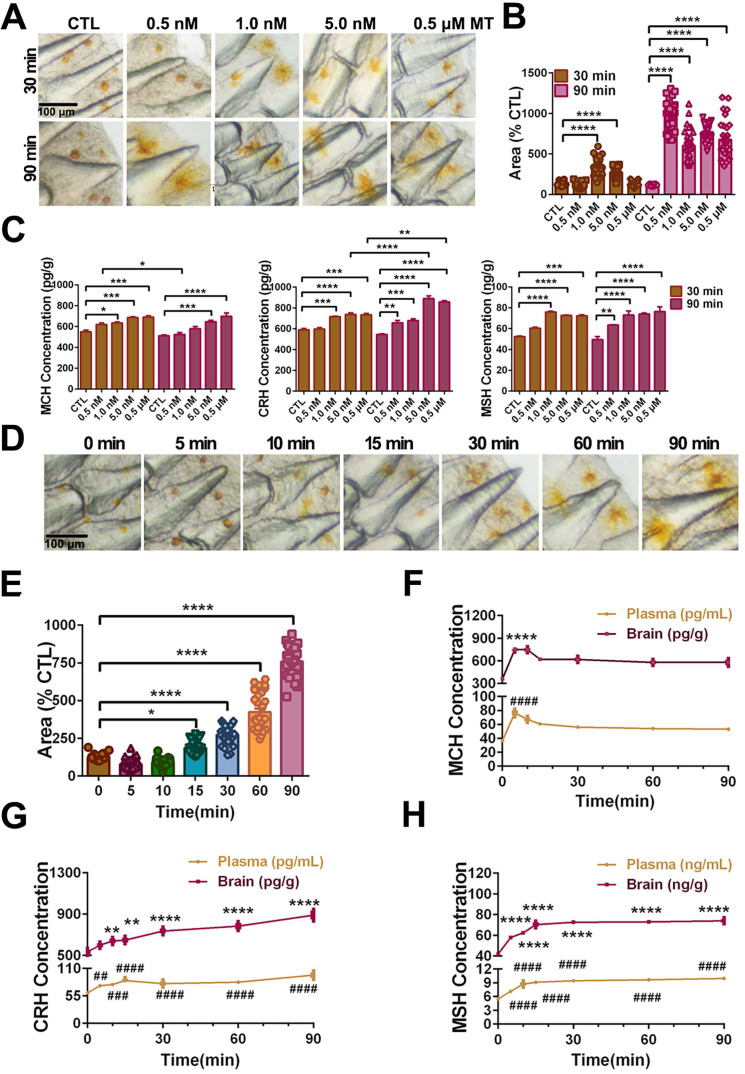
**MT-mediated regulation of pigment granule mobilization and neuropeptides secretion in the large yellow croaker *in vivo*.** *(A)* Microphotographs showing pigment granule mobilization in the xanthophore exposed to different concentrations of melatonin (MT) for 30 min and 90 min, respectively. *(B)* Bar chart showing the areas of xanthophore covering the ventral skin after 30 or 90 min treatment with different concentrations of MT. *(C)* Variation in brain melanin-concentrating hormone (MCH), corticotropin releasing hormone (CRH) and melanocyte-stimulating hormone (MSH) secretion after 30 min or 90 min treatment with different concentrations of MT. *(D)* Microphotographs showing time-dependent pigment granule mobilization in the xanthophore exposed to 5.0 nM MT for 90 min. The results are expressed as areas of xanthophores relative to nontreated groups *(B and D)*.* (E)* Bar chart showing the areas of xanthophore covering the ventral skin after 90 min treatment with 5.0 nM MT. *(F)* The time-dependent changes in the plasma and brain MCH, CRH and MSH levels after 90 min treatment with 5.0 nM MT. Each data represents mean (± SEM) (n=3). The results are expressed as MCH, CRH and MSH concentration relative to nontreated groups *(C,F-H)*.Data were analyzed using the one-way ANOVA followed by Tukey's multiple comparison test, * *P* < 0.05, ** P < 0.01, *** P < 0.001, **** P < 0.0001, ^#^ *P* < 0.05, ^##^* P* < 0.01, ^###^* P* < 0.001, ^####^* P* < 0.0001. MCH: Melanin-concentrating hormone; CRH: Corticotropin-releasing hormone; MSH: melanocyte-stimulating hormone.

**Pharmacological effects of MT on the pigment granule mobilization through MT/MCH and MT/(TAC1+CRH)/MSH signaling pathway.** To determine the effect of MT on the skin color changes, we injected different concentrations of MT following a time course during the day (light intensity: 81.3 ± 1.2 lx). After different doses of MT for 30-90 min treatment, large extensions to the areas of xanthophores covering the ventral skin were observed (Fig. 8A). Compared to the control, the dispersion of xanthophores significantly increased at 1.0 nM MT and 5.0 nM MT with a peak level at 1.0 nM after 30 min, while in the 90 min group, the peak value of xanthophore dispersion was observed at the lower dose (0.5 nM) (Fig. 8A-B). Meanwhile, the brain MCH, CRH, and MSH levels were enhanced in response to MT administration in a dose-dependent manner (Fig. 8C). Furthermore, the effect of 5.0 nM MT administration was evaluated over a time course. We found that the area of ventral xanthophores slightly decreased at 5 and 10 min, then significantly increased in a time-dependent manner after 15 min (Fig. 8D-E), and the melanocytes of dorsal skin aggregated after MT treatment for 5 min and gradually dispersed later at 15 min (Fig. S22). Interestingly, time-dependent elevation in CRH and MSH concentrations were determined in the plasma and brain, while the MT-induced MCH secretion was rapid (with peak value at 5 min) and returned to almost basal levels within 90 min (Fig. 8F-H). The results of dorsal and ventral skin coloration quantified by the chromatometer were consistent with the above findings (Fig. S23). In addition, *in vitro* MT (0.25 μM) treatment did not significantly enhance the b* values of the ventral skin of large yellow croaker (Fig. S24).

To further evaluate neuropeptide responses to MT administration, we measured ERK phosphorylation and neuropeptide expression in MT-treated hypothalamus tissue *in vitro*. *Lc*Mtnrs activation significantly induced ERK phosphorylation and the secretion of MCH, TAC1 and CRH in the hypothalamic tissues (significant decreases in neuropeptide level were detected in cultured tissue samples, see Fig. S25A). Furthermore, mature peptides CRH, Neurokinin A (NKA) or SP significantly elevated α-MSH level in the pituitary *in vitro* (Fig. S25B). Collectively, both *in vivo* and *in vitro* results suggest that MT/MCH and MT/(TAC1+CRH)/MSH signaling pathways are involved in chromatophore mobilization.

**Figure 9:**
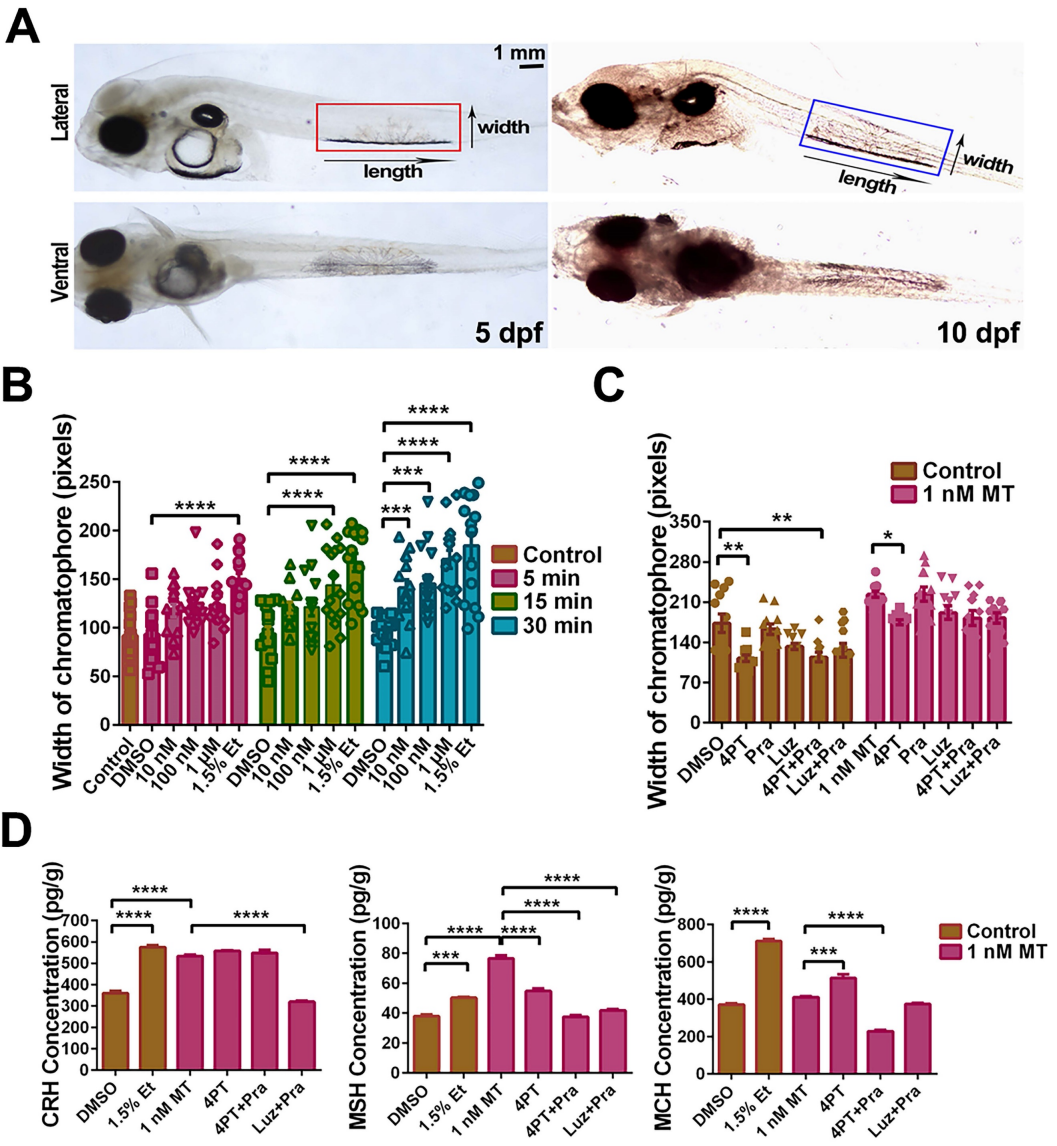
**MT-mediated regulation of pigment cell width and neuropeptide secretion in the large yellow croaker larvae *in vitro*.** *(A)* Microphotographs from dorsal and lateral view of 5 and 10 dpf larvae. Pigment cells are marked by boxes and the width and length of pigment cells are indicated by an arrow. Scale bar: 1 mm. *(B)* The bar chart shows the width of the chromatophore after incubation with different concentrations of MT for 5, 15 or 30 min, and control (1.5% ethanol). *(C)* Bar chart showing the width of the chromatophore pre-treated with 50 μM 4-P-PDOT (4PT), 50 μM prazosin hydrochloride (Pra), 50 μM luzindole (Luz), 50 μM 4PT+50 μM Pra and 50 μM Luz+50 μM Pra for 2 h first and then incubated with 1 nM MT for an additional 30 min. *(D)* The changes in larvae melanin-concentrating hormone (MCH), corticotropin releasing hormone (CRH) and melanocyte-stimulating hormone (MSH) contents after 30 min incubation with 1.5% ethanol or 2 h pretreatment with 50 μM 4PT, 50 μM 4PT+50 μM Pra and 50 μM Luz+50 μM Pra and then 30 min incubation with 1 nM MT. Data were analyzed as means ± SEM (n=3) using the one-way ANOVA followed by Tukey's multiple comparison test, *P < 0.05, ** P < 0.01, *** P < 0.001, **** P < 0.0001.

***Lc*Mtnr1b subtypes contribute to neuroendocrine control of pigment granule mobilization.** After verifying the expression of *Lcmtnrs* in larvae (Fig. S26), we defined the length and width of pigment cells (Fig. 9A), after incubation with different concentrations of MT for 5, 15, or 30 min, with 1.5% ethanol as a positive control (Fig. 9B). We also used different antagonists for melatonin receptors to pharmacologically block MT signaling. After incubation with 1 nM MT for 30 min, the chromatophore width and the levels of CRH, MSH, and MCH increased, which was consistent with the results from adult fish. Such activity was significantly inhibited by the *mtnr1b* antagonist 4-P-PDOT (4PT) (Fig. 9C-D). The effects of Mtnrs-antagonists on *Lc*Mtnrs were pre-tested (Fig. S27). These data suggest that *Lc*Mtnr1b contributes to the neuroendocrine control of pigment granule mobilization.

## Discussion

MT, the neuro-hormone mainly produced by the pineal gland, is responsible for various physiological activities in vertebrates, especially for circadian rhythms. Its multiple effects are attributable to the diversity of MT targets, whose functions are poorly understood. The high-affinity membrane receptors represent most of the functional MT mediators. Herein, we systematically characterized all five Mtnrs in a marine teleost species, large yellow croaker, and further uncovered the functional role of MT in the central neuroendocrine system. We found that MT regulates camouflage in fish through its function in the MT/MCH and MT/(TAC1+CRH)/POMC pathways. To our knowledge, this is the first in depth investigation of the MT signaling system in regulating the hypothalamus-pituitary axis, especially in the context of chromatophore mobilization control.

Our data reveal two Mtnr1a subtypes, two Mtnr1b subtypes, and one Mtnr1c subtype in the large yellow croaker. Similar to Mtnrs in other species, all five *Lc*Mtnrs belong to GPCR classA/1 (or rhodopsin-like receptors). Mammalian Mtnr1a and Mtnr1b signal primarily via G_αi_ proteins 49, 50. The G_αs_ protein signaling cascade can also be activated by specific fish Mtnr subtypes 51, but G_αq_ protein-coupled signaling pattern still needs to be clarified in teleost species. Herein, intracellular signal transduction mediated by all five obtained *Lc*Mtnrs was systematically elucidated. The results showed that only one Mtnr1a subtype (*Lc*Mtnr1a2) and* Lc*Mtnr1c activated G_αi_ protein coupled MAPK signaling pathway. Interestingly, another Mtnr1a subtype, *Lc*Mtnr1a1, was found to be dual-coupled with G_αi_/G_αs_ proteins. This is consistent with previous findings in human Mtnr1a 52, indicating that coupling of Mtnr1a to different signal transduction cascades may be evolutionarily conserved, and that the diversity of signaling pathways of different Mtnr1a subtypes may contribute to the complexity of Mtnrs functions in fish. Furthermore, our results showed no significant cAMP variations mediated by the two activated-*Lc*Mtnr1b paralogs, at least in the HEK293 cell line, indicating that G_αi_ or G_αs_ protein is not likely to play a part in both* Lc*Mtnr1b1- and *Lc*Mtnr1b2-induced downstream signals, which is inconsistent with the results of a Mtnr1b by Chen et al 53. Both activated-*Lc*Mtnr1b proteins have been determined to trigger the accumulation of intracellular Ca^2+^ in HEK293 cells. This is the first evidence to our knowledge that fish Mtnr1b activates the G_αq_ coupling signaling pathway, which has only been reported in mammals and birds (e.g. ovine pars tuberalis 54 and rat myometrium 55.

The effects of MT are mediated through complex interactions with the hypothalamic and pituitary neuroendocrine cells. For example, pituitary luteinizing hormone (LH) release can be decreased by intraventricular administration of MT in the preoptic area, affecting reproduction in the Atlantic croaker (*Micropogonias undulatus*) 11. The transcript levels of *kisspeptin* (*kiss1* and *kiss2*), *gonadotropin-releasing hormone 1* (*gnrh1*), and the *β-subunit of gonadotropins* (*fshβ* and *lhβ*) in the brain of the sapphire devil can be significantly inhibited by long photoperiod or melatonin administration affecting its reproductive activity 56. And in freshwater eels, the MT was presented as upstream modulator functioning on all levels of the brain-pituitary-gonad (BPG) axis reflecting the effects from external cues 57. In mammals, MT also acts in concert with neurotransmitters such as γ-aminobutyric acid, dopamine, and glutamate in the central nervous system and controls body movement, reproduction, and other neurotransmitter-related functions 58, 59. These functional patterns of the MT signaling system have also been discovered in zebrafish 60, 61, but a comprehensive overview of how the MT signaling system acts on the neuroendocrine center (hypothalamus and pituitary) in teleosts is not well understood. Herein, we performed single-cell RNA sequencing to explore the transcriptional content of five *Lc*Mtnrs and neuropeptides within individual cells derived from the hypothalamus and pituitary in the large yellow croaker. Our data showed that hypothalamic cells were initially clustered into neural and non-neural cell populations based on the expression of the pan-neuronal marker genes *Syt1* and *Snap25*, which is generally consistent with the reported findings in mice 48. All five *Lcmtnrs* were highly expressed in neuronal cells, and distinct transcriptional levels of *Lcmtnrs* were detected in GABAergic, dopaminergic, and glutamatergic neurons. The evidence collected from *in silico* and experimental approaches further demonstrates that the MT signaling system may be an upstream regulator of hypothalamus *Pmch*^+^, *Pmchl*^+^, *Tac1*^+^, *Crh1*^+^, and pituitary *Pomc*^+^ (precursor of MSH) neurons. More importantly, our study has constructed, for the first time, the signal patterns of the MT signaling system in the neuroendocrine center, suggesting a potential role of MT in neuroendocrine functional regulation in the large yellow croaker, specifically in energy balance, metabolic regulation, and chromatophore mobilization.

In zebrafish larvae, camouflage involves MCH and α-MSH in promoting melanosome dispersal or aggregation in melanocytes and contains a critical CRH-POMC pathway 34. A recent study in large yellow croaker revealed that xanthophore dispersion also involves MSH 31. These data suggest that camouflage is conserved among different teleosts. MT has known pigment dispersion effects in fish species, although it is generally considered an aggregation inducer 62, 37. This raises an important scientific question as to whether MT signaling systems act in this complex regulatory network and what role they play in regulating the camouflage behavior of fish. Through pharmacological experiments, we showed that MT treatment triggered xanthosome aggregation and remarkably enhanced MCH production within 5 min, resulting in an increase in silver white ventral body coloration in the large yellow croaker. To our surprise, with the time course of MT treatment, the dispersion of xanthosomes was observed gradually, and the MCH level declined with the concomitantly elevated levels of CRH and MSH, resulting in golden yellow coloration of the fish ventral body after the MT treatment of 30 min. These results suggest that MT is an upstream regulator of xanthosome mobilization acting on both MCH and CRH-MSH, while the effect on MCH appears first, possibly in a MT concentration-dependent manner and through engaging different Mtnrs.

*Tac1* is expressed in numerous neurons of mammalian brains, and the tachykinins interact with kisspeptin, α-MSH and other neuroendocrine factors, in puberty control 63. The tachykinins are also activated by long photoperiod, and defined as seasonal breeding controllers in addition to the thyrotropin beta subunit (TSHβ), the transcriptional cofactor eyes absent 3 (Eya3), as well as melatonin 64, 65. Our results from scRNA-seq and experimental data collectively revealed a signaling pathway MT/TAC1/MSH, parallel to MT/CRH/MSH, and indicated that TAC1 can be activated by melatonin administration and subsequently triggers the high expression of α-MSH. This enriches the regulatory functions of MT in photoperiod derived neuroendocrine signals.

The *mtnr1a* and *mtnr1b* transcripts has been reported in both neural and peripheral tissues of mammals 66 and fish 21, suggesting their involvement in various biological processes. However, *mtnr1c* mRNA expression appears to be restricted to neural tissues (the brain and retina) 23. Our study revealed that all five *Lcmtnrs* are expressed in neural and peripheral tissues (especially in the brain and head kidney), as reported in human 67 and reef fish kidney 21, suggesting that these five *Lc*Mtnrs subtypes play functional roles in neural and immune responses. Additionally, a wider expression distribution of* Lcmtnr1b2* in the heart and intestine was observed compared to *Lcmtnr1b1*, which showed differential expression patterns of *mtnr1b*-paralogs, suggesting that each *Lc*Mtnr paralog has distinct functions. Thus, all five *Lc*Mtnrs play important roles in neural and immune responses, but have distinct functions in peripheral tissues.

In conclusion, our findings provide a better understanding of the signal transduction pathways involving MT, although the signals affected by dimerization of *Lc*Mtnrs are not evaluated in the current study. Through an in-depth analysis of single-cell transcriptomic data, we propose an interaction model between MT and neuropeptidergic neurons (mainly *Pmch^+^*, *Tac1*^+^, *Crh*^+^ and *Pomc^+^* neurons). The model was further verified to show that MT plays a functional role in TAC1-MSH, CRH-MSH and MCH (PMCH-derived) control in the large yellow croaker. Importantly, we reveal a new skin color adaptation strategy in teleosts regulated by the MT signaling system, mainly by the Mtnr1b subtype. Collectively, through systematic studies of MT signaling in a non-conventional fish, this work establishes that the plasticity of the MT signaling system likely contributes to variations in environmental adaptations and other neuroendocrine functions among vertebrates.

## Supplementary Material

Supplementary figures and tables, data.

## Abbreviations

5-HT: 5-hydroxytryptamine (serotonin)
AANAT: Alkylamine N-acetyltransferase
AC: Adenylate Cyclase
ACBD7: Acyl-CoA Binding Domain Containing 7
Adcyap1: adenylate cyclase activating polypeptide 1
Adm: adrenomedullin
Apln: apelin
BF: Bright Field
BPG: brain-pituitary-gonad
Calm: Calmodulin
cAMP: Cyclic adenylic acid
Cbln2: cerebellin 2 precursor
CRH: Corticotropin-releasing hormone
DAG: Diacylglycerol
ERK1/2: Extracellular signal-regulated kinase 1/2
Gad1: glutamate decarboxylase 1
Gad2: glutamate decarboxylase 2
GnRH: gonadotropin-releasing hormone
GPCR: G protein-coupled receptor
Gria2: glutamate ionotropic receptor AMPA type subunit 2
Grid2: glutamate ionotropic receptor delta type subunit 2
Hd: dorsal zones of the hypothalamus
HPG: Hypothalamo-pituitary-gonadal
HPT: Hypothalamic-pituitary-thyroid
Hv: ventral zones of the hypothalamus
Igf: insulin-like growth factor
Ins: insulin
IP3: Inositol triphosphate
Ist: IST1 factor associated with ESCRT-III
Kiss: kisspeptin
Lmx1b: LIM homeobox transcription factor 1 beta
LOF: Large yellow croaker ovary fibroblast
Luz: Luzindole
MAPK: Mitogen activated protein kinase
MCH: Melanin-concentrating hormone
MSH: Melanocyte-stimulating hormone
MT: Melatonin
Mtnrs: melatonin receptors
NKA: Neurokinin A
NPY: neuropeptide Y
NPY: Neuropeptide Y
P: phosphorylation
PI: pars intermedia
PKA: Protein Kinase A
PKC: Protein Kinase C
PLC: Phospholipase C
Pmch: pro-melanin concentrating hormone
Pmchl: pro-melanin concentrating hormone, like
PN: neurohypophysis
Pomc: proopiomelanocortin
PPD: proximal pars distalis
Pra: prazosin hydrochloride
PTX: pertussis toxin
RPD: rostral pars distalis
Scg2: secretogranin II
Scg5: secretogranin V
Slc17a6: solute carrier family 17 member 6
Slc32a1: solute carrier family 32 member 1
Snap25: synaptosome associated protein 25
SP: Substance P
Sv: Saccus vasulosus
Syt1: synaptotagmin 1
TAC1: Tachykinin precursor 1
Vgf: VGF nerve growth factor inducible
