# Supplementary material for: Melatonin modulates the hypothalamic-pituitary neuroendocrine axis to regulate physiological color change in teleost fish
Source: Int J Biol Sci. 2023 Jun 4;19(9):2914–33. doi: 10.7150/ijbs.81055 (PMC10266084; doi:10.7150/ijbs.81055)
Supplement: Supplementary file 1 — Supplementary figures and tables, data. [file ijbsv19p2914s1.pdf]

Supplementary information for

Melatonin modulates the hypothalamic-pituitary neuroendocrine axis to regulate physiological color change in teleost fish

Jiaqian Feng[1#], Jingwen Yang[1#*], Zhijing Jiang[1#], Naiming Zhou[2], Xue Liu[1], Guangbo Zhang[1], Xiaojun Yan[1], Jixiu Wang[1], Xiuwen Xu[1], Su Guo[3*], Tianming Wang[1*]

[1]National Engineering Laboratory of Marine Germplasm Resources Exploration and Utilization, Marine Science College, Zhejiang Ocean University, Zhoushan, Zhejiang 316022, People's Republic of China

[2]Institute of Biochemistry, College of Life Sciences, Zijingang Campus, Zhejiang University, Hangzhou, Zhejiang 310058, People's Republic of China

[3]Programs in Human Genetics and Biological Sciences, Department of Bioengineering and Therapeutic Sciences, University of California, San Francisco, San Francisco, CA 94158 , United States

[#]These authors contributed equally

*Corresponding author: Dr. Jingwen Yang (yangjingwen@zjou.edu.cn); Dr. Su Guo (Su.Guo@ucsf.edu); Dr. Tianming Wang (wangtianming@zjou.edu.cn)

This PDF file includes:

Figures S1–27

Table S1-3

Other supplementary materials for this manuscript include the following:

Source data 1-3 for Figure 5 and Figure S17-18

Figures

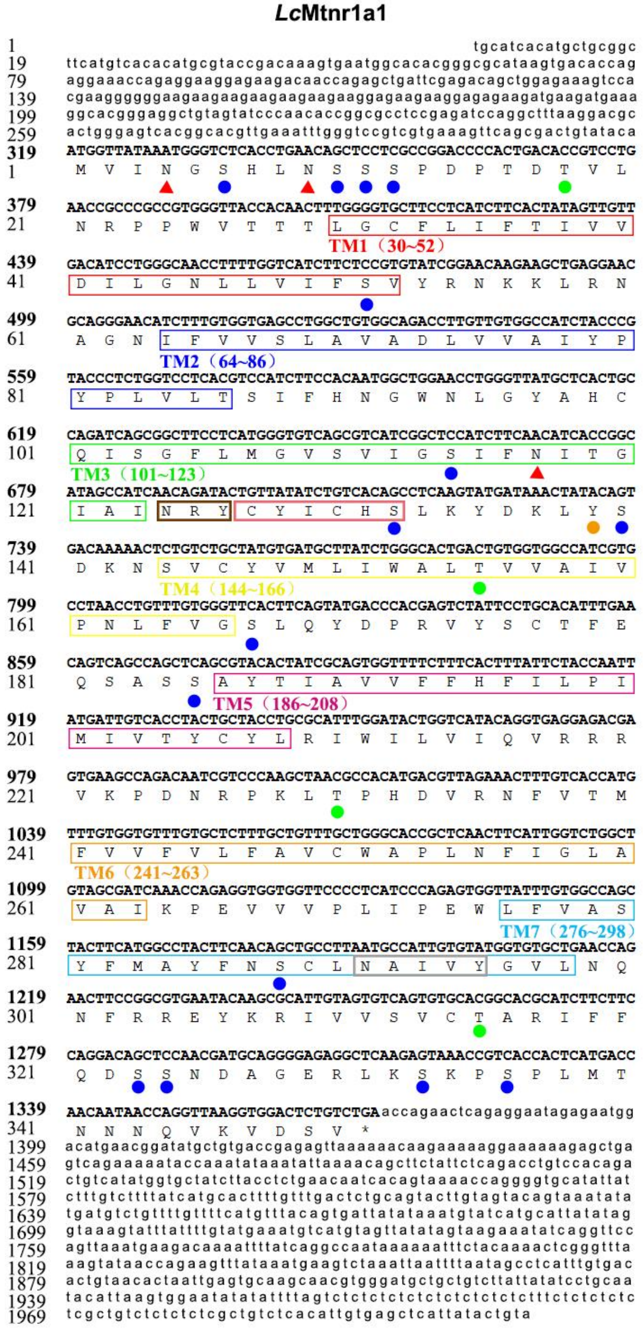

Fig. S1. Characteristics of gene structure and the deduced amino acid sequence of *Lc*Mtnr1a1-like.The phosphorylation sites predicted with NetPhos 3.1 Server are marked by solid dots in three difficult colors (Ser, Tyr and green are in blue, orange and green, respectively); the N-linked glycosylation sites predicted with NetNGlyc 1.0 Server are highlighted by red triangles; Seven-transmembrane (7TM) domains predicted with I-TASSER are labeled in boxes in 7 different colors; the NRY, CYICHS and NAXXY sequences predicted with PROSITE are labeled in brown, pink and gray boxes, respectively. TM: transmembrane.

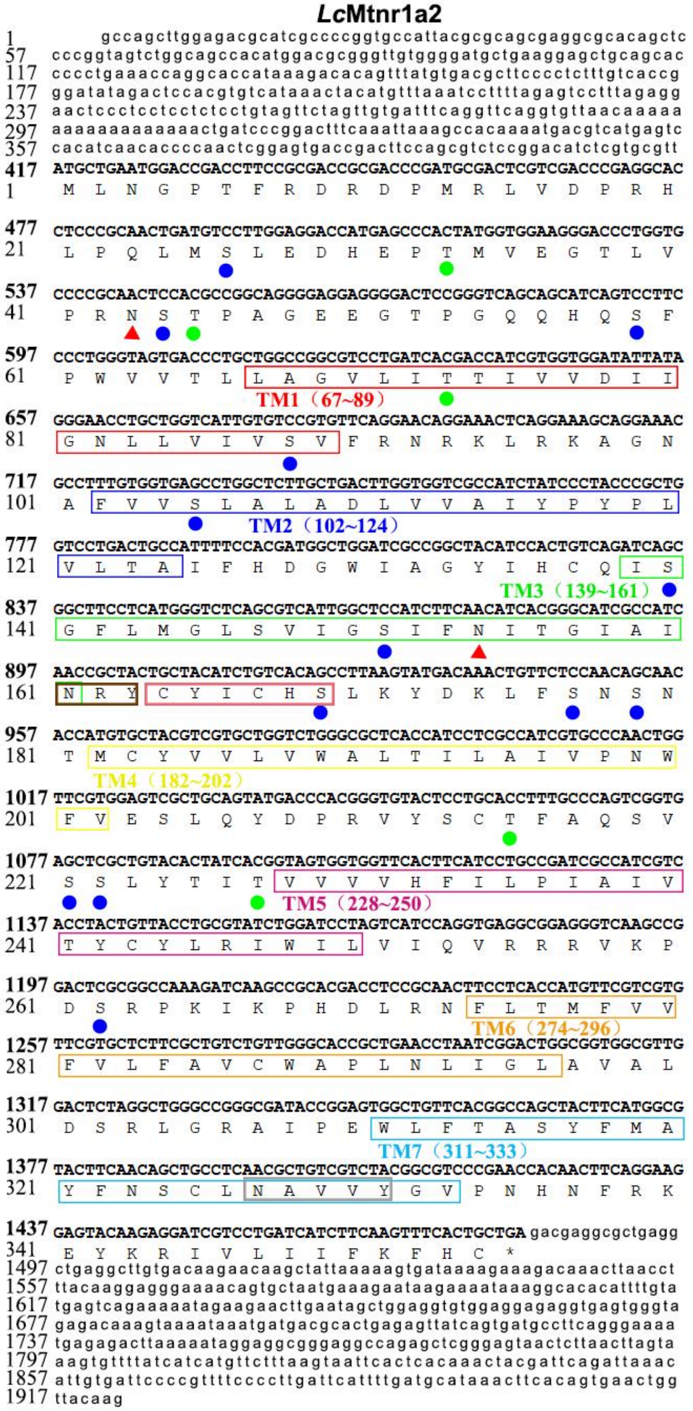

Figure S2. Characteristics of gene structure and the deduced amino acid sequence of *Lc*Mtnr1a2-like.The phosphorylation sites predicted with NetPhos 3.1 Server are marked by solid dots in three difficult colors (Ser, Tyr and green are in blue, orange and green, respectively); the N-linked glycosylation sites predicted with NetNGlyc 1.0 Server are highlighted by red triangles; 7TM domains predicted with I-TASSER are labeled in boxes in 7 different colors; the NRY, CYICHS and NAXXY sequences predicted with PROSITE are labeled in brown, pink and gray boxes, respectively. TM: transmembrane.

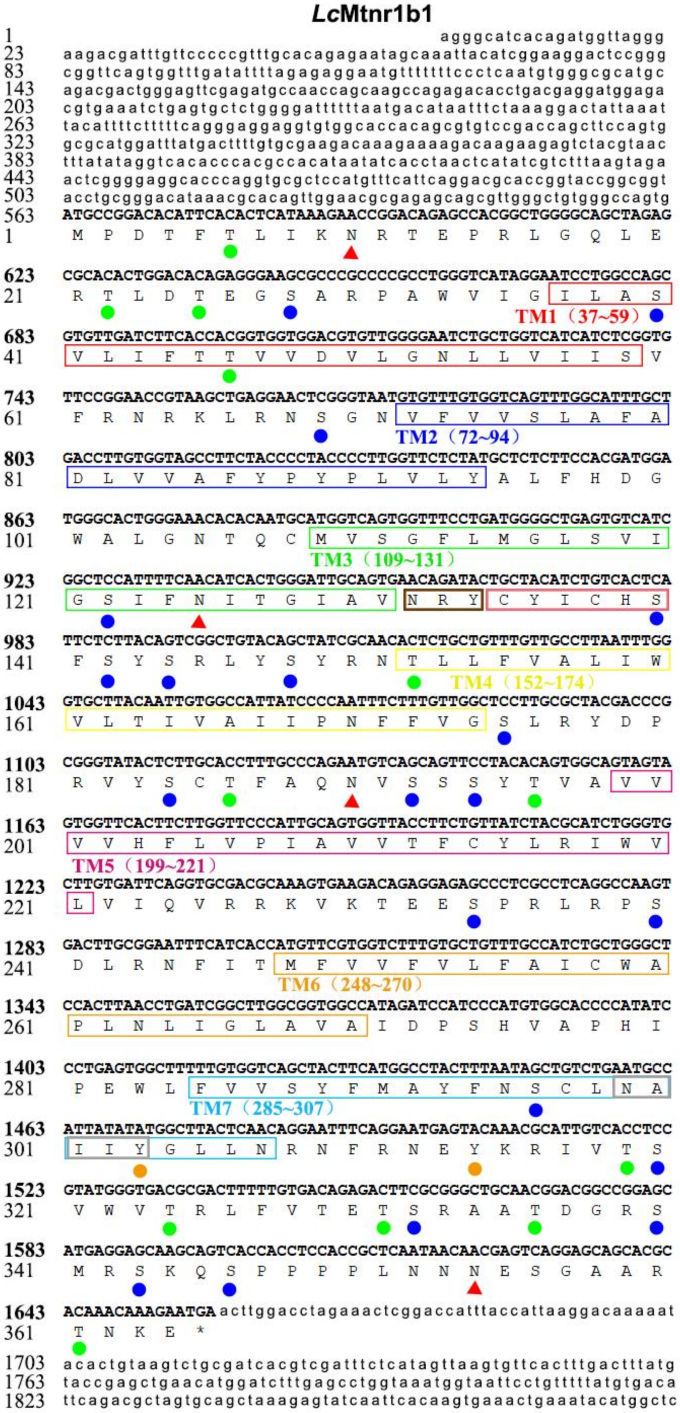

Figure S3. Characteristics of gene structure and the deduced amino acid sequence of *Lc*Mtnr1b1-like. The phosphorylation sites predicted with NetPhos 3.1 Server are marked by solid dots in three difficult colors (Ser, Tyr and green are in blue, orange and green, respectively); the N-linked glycosylation sites predicted with NetNGlyc 1.0 Server are highlighted by red triangles; 7TM domains predicted with I-TASSER are labeled in boxes in 7 different colors; the NRY, CYICHS and NAXXY sequences predicted with PROSITE are labeled in brown, pink and gray boxes, respectively. TM: transmembrane.

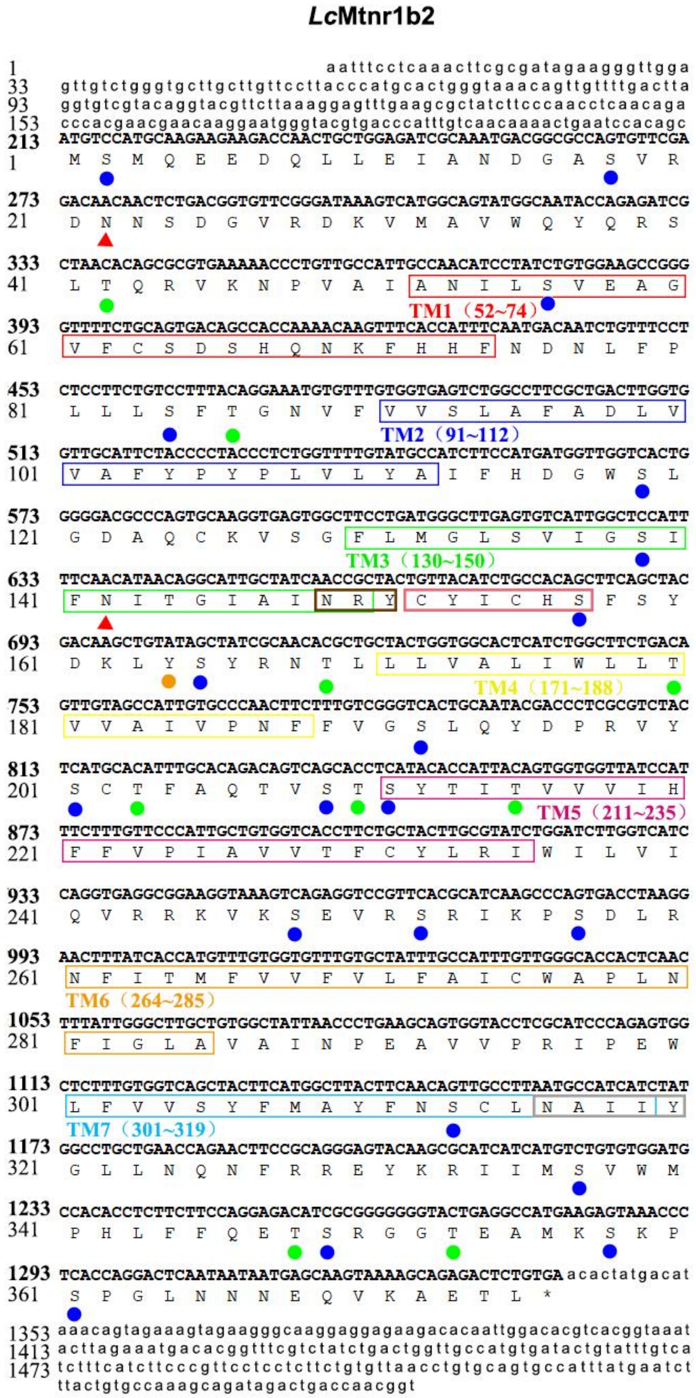

Figure S4. Characteristics of gene structure and the deduced amino acid sequence of *Lc*Mtnr1b2-like.The phosphorylation sites predicted with NetPhos 3.1 Server are marked by solid dots in three difficult colors (Ser, Tyr and green are in blue, orange and green, respectively); the N-linked glycosylation sites predicted with NetNGlyc 1.0 Server are highlighted by red triangles; 7TM domains predicted with I-TASSER are labeled in the boxes in 7 different colors; the NRY, CYICHS and NAXXY sequences predicted with PROSITE are labeled in brown, pink and gray boxes, respectively. TM: transmembrane.

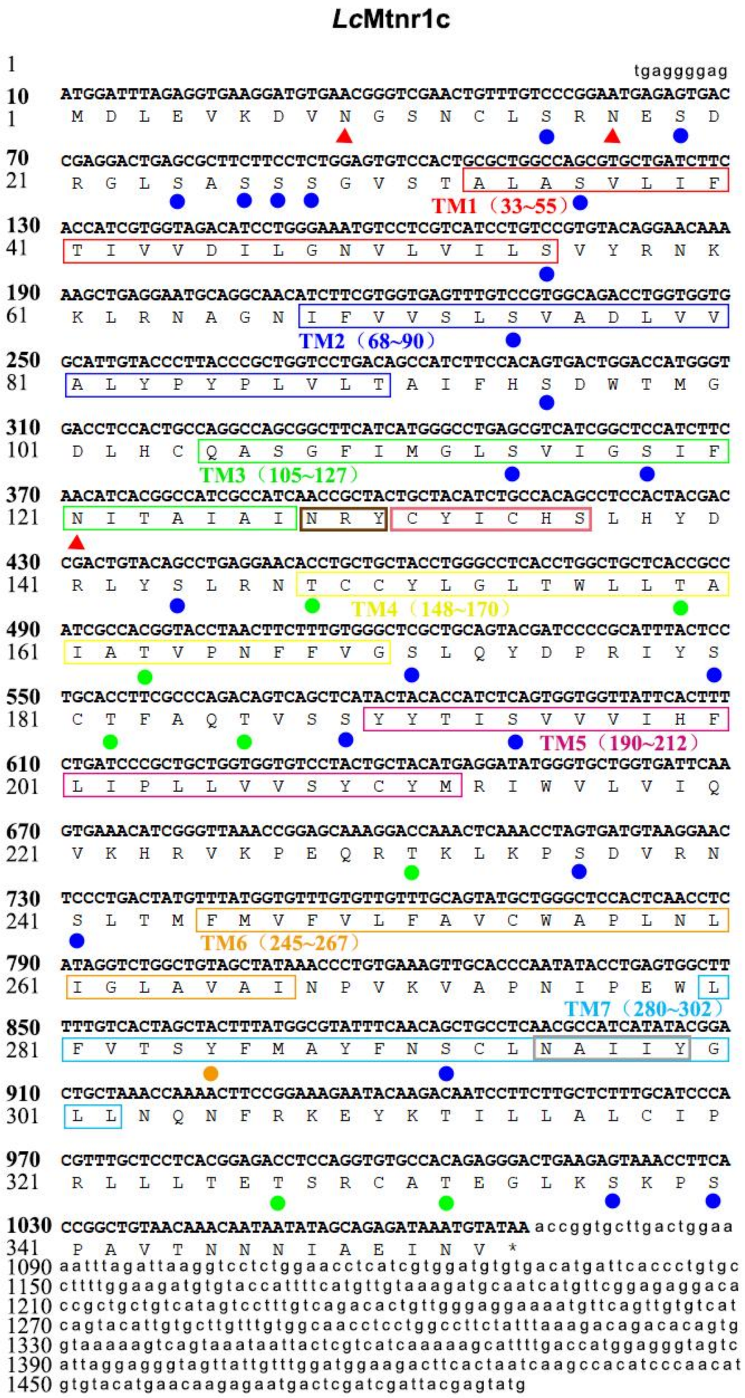

Figure S5. Characteristics of gene structure and the deduced amino acid sequence of *Lc*Mtnr1c-like. The phosphorylation sites predicted with NetPhos 3.1 Server are marked by solid dots in three difficult colors (Ser, Tyr and green are in blue, orange and green, respectively); the N-linked glycosylation sites predicted with NetNGlyc 1.0 Server are highlighted by red triangles; 7TM domains predicted with I-TASSER are labeled in the boxes in 7 different colors; the NRY, CYICHS and NAXXY sequences predicted with PROSITE are labeled in brown, pink and gray boxes, respectively. TM: transmembrane.

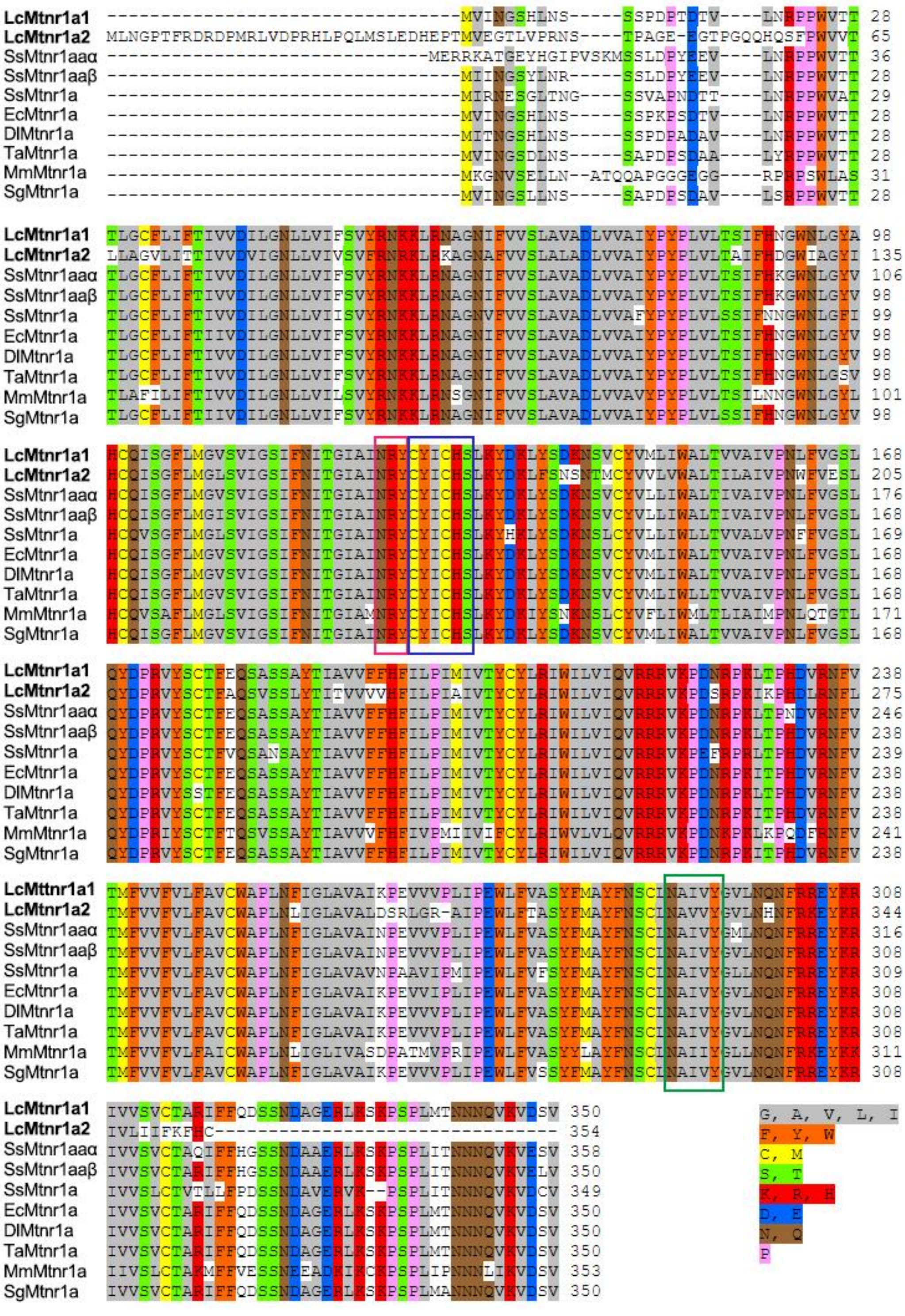

Figure S6. Alignment of the deduced *Lc*Mtnr1a amino-acid sequences with vertebrate Mtnr1a from other species. All sequences were obtained from GenBank and their accession numbers are listed in Table S1. The NRY, CYICHS and NAXXY sequences are marked with pink, dark blue and green boxes, respectively. The percentage of sequences that must agree for identity or similarity coloring was set as 80%.

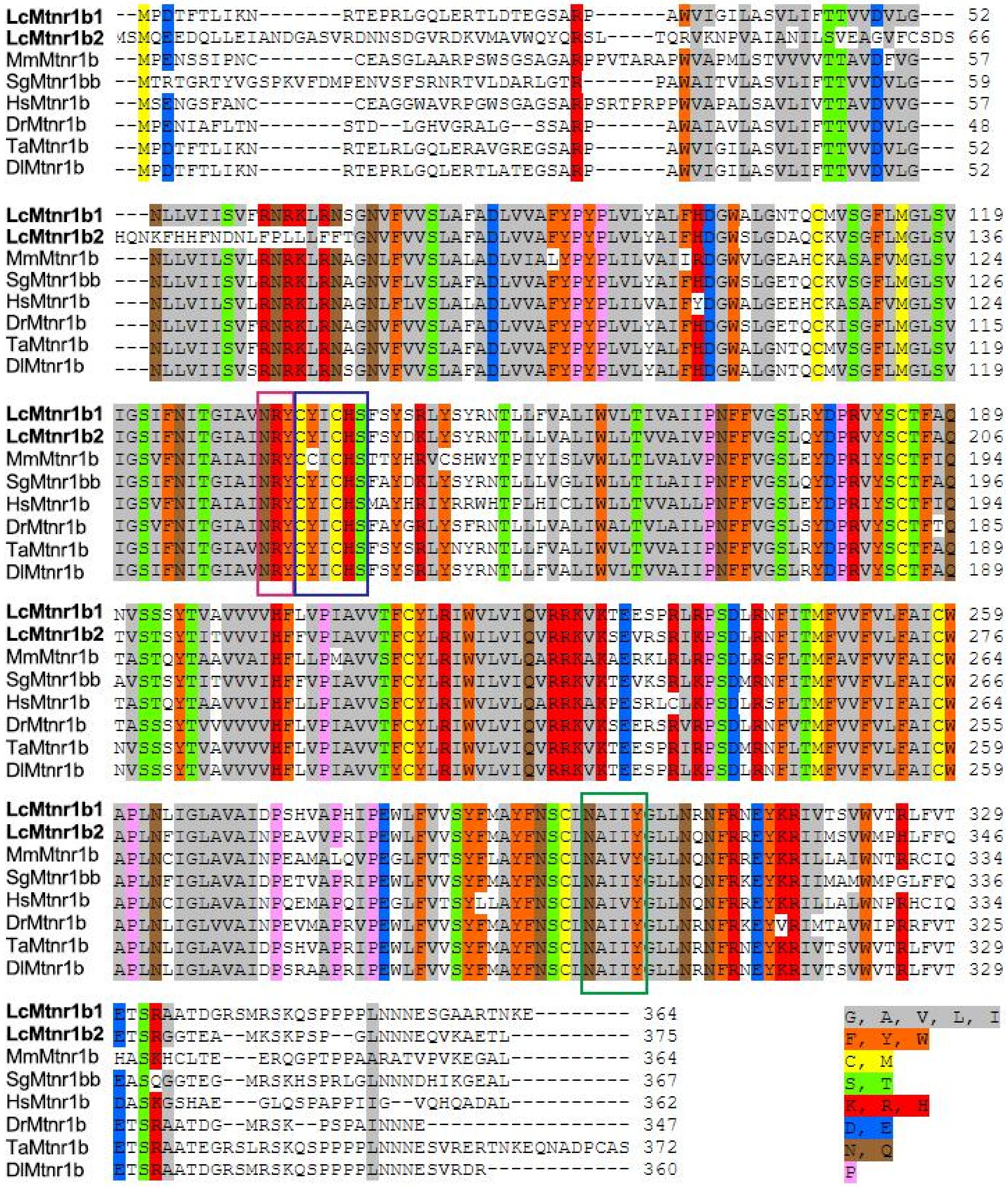

Figure S7. Alignment of the deduced *Lc*Mtnr1b amino-acid sequences with vertebrate Mtnr1a from other species. All sequences were obtained from GenBank and their accession numbers are listed in Table S1. The NRY, CYICHS and NAXXY sequences are marked with pink, dark blue and green boxes, respectively. The percentage of sequences that must agree for identity or similarity coloring was set as 80%.

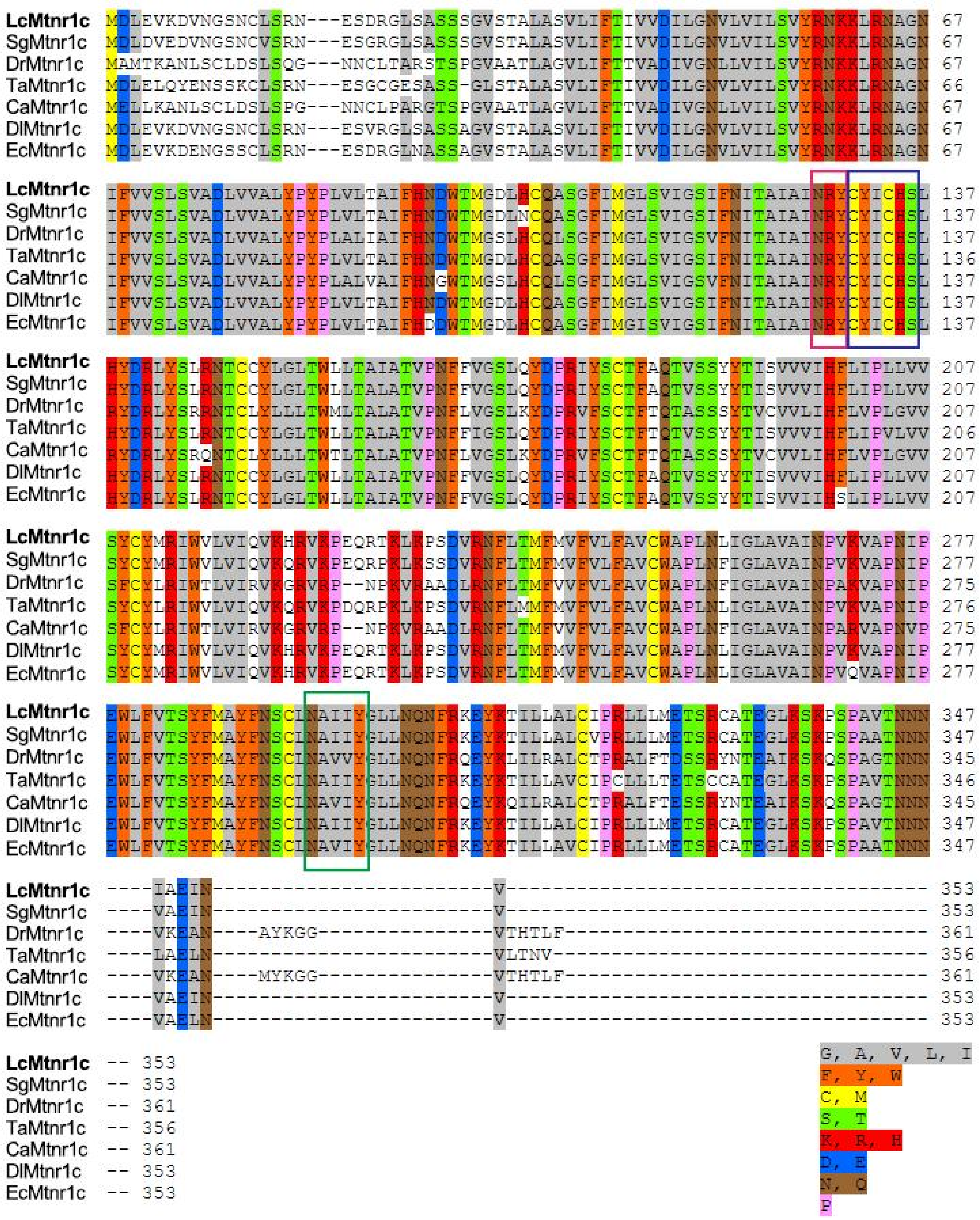

Figure S8. Alignment of the deduced *Lc*Mtnr1c amino-acid sequences with vertebrate Mtnr1a from other species. All sequences were obtained from GenBank and their accession numbers are listed in Table S1. The NRY, CYICHS and NAXXY sequences are marked with pink, dark blue and green boxes, respectively. The percentage of sequences that must agree for identity or similarity coloring was set as 80%.

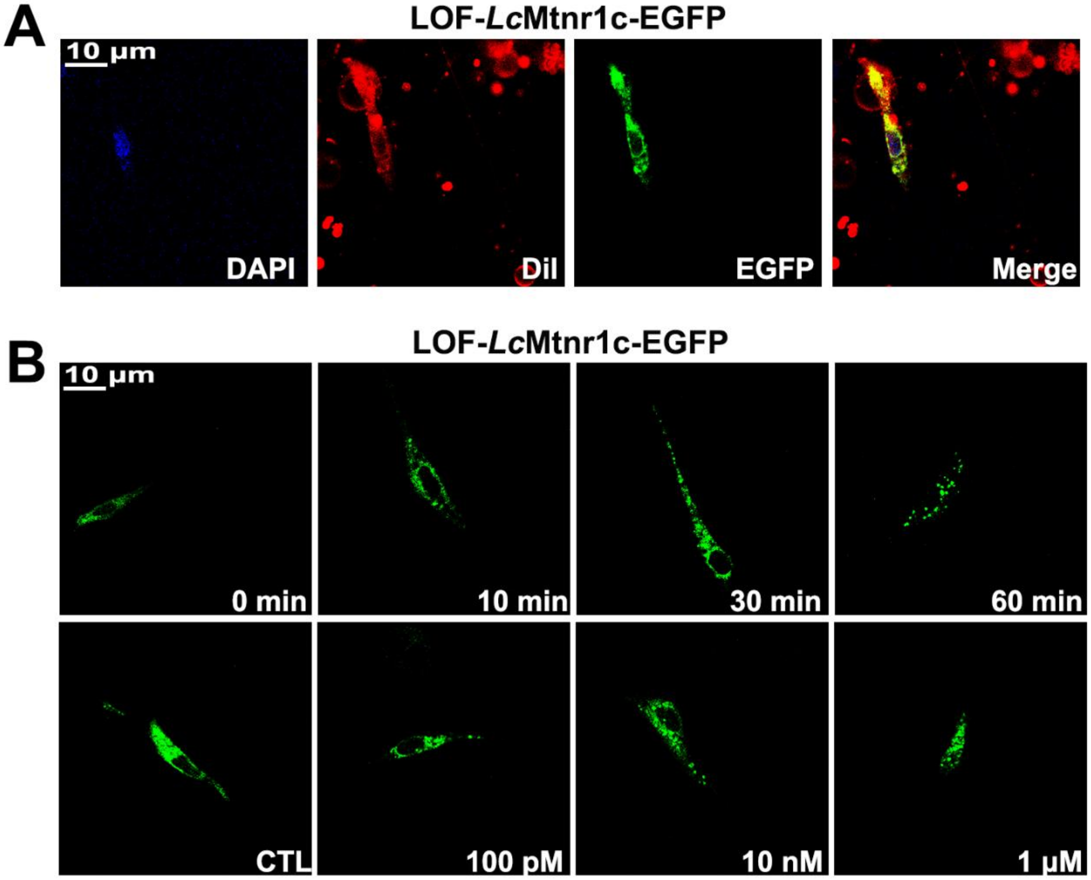

Figure S9. Characterization of *Lc*Mtnr1c-EGFP stably expressed in large yellow croaker ovary fibroblast (LOF) cells. (A) Subcellular location of *Lc*Mtnr1c-EGFP in large yellow croaker ovary fibroblast (LOF) cells. LOF cells stably expressing *Lc*Mtnr1c-EGFP were stained with a membrane plasma probe (Dil, 5 μM) and a nuclei probe (DAPI). (B) Internalization of *Lc*Mtnr1c-EGFP stably expressed in LOF cells. LOF-*Lc*Mtnr1c cells were incubated with 100 nM MT for the indicated time periods or indicated concentrations of MT at 37 °C for 60 min. Cells were fixed and examined with confocal microscopy as described under "Methods". All pictures shown are representative of at least three independent experiments.

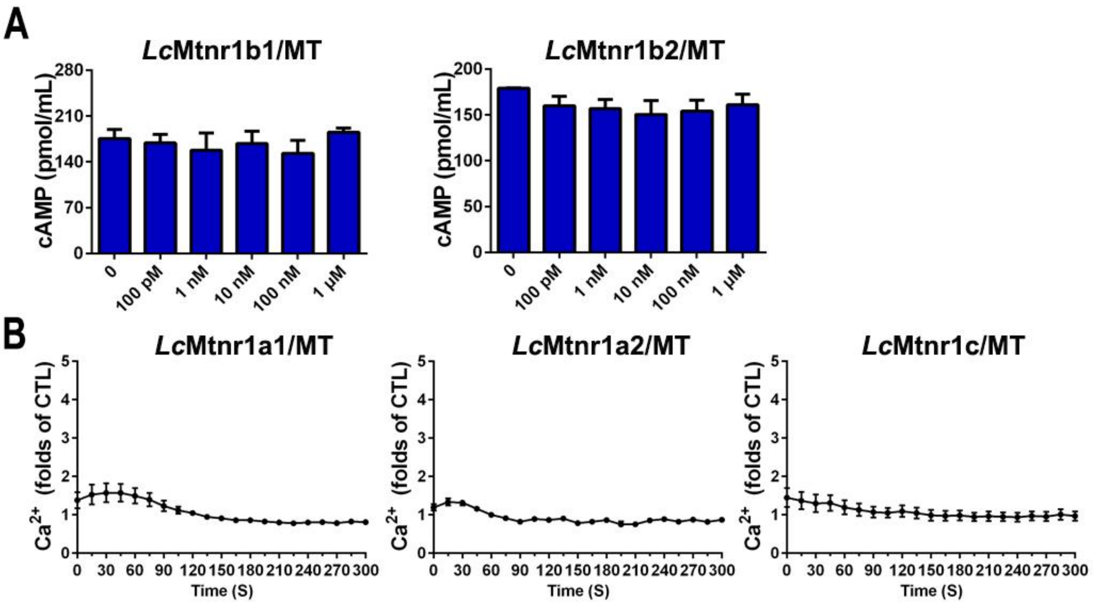

Figure S10. The detection of cAMP accumulation and Ca^{2+} mobilization in MT activated *Lc*Mtnr1b1/2 or *Lc*Mtnr1a1/1a2/1c expressing cells. (A) cAMP accumulation in *Lc*Mtnr1b1/2-expressing HEK293 cells was measured in response to the indicated concentration of MT for 15 min. (B) Ca^{2+} mobilization in *Lc*Mtnr1a1/1a2/1c-expressing HEK293 cells was measured in response to 100 nM MT.

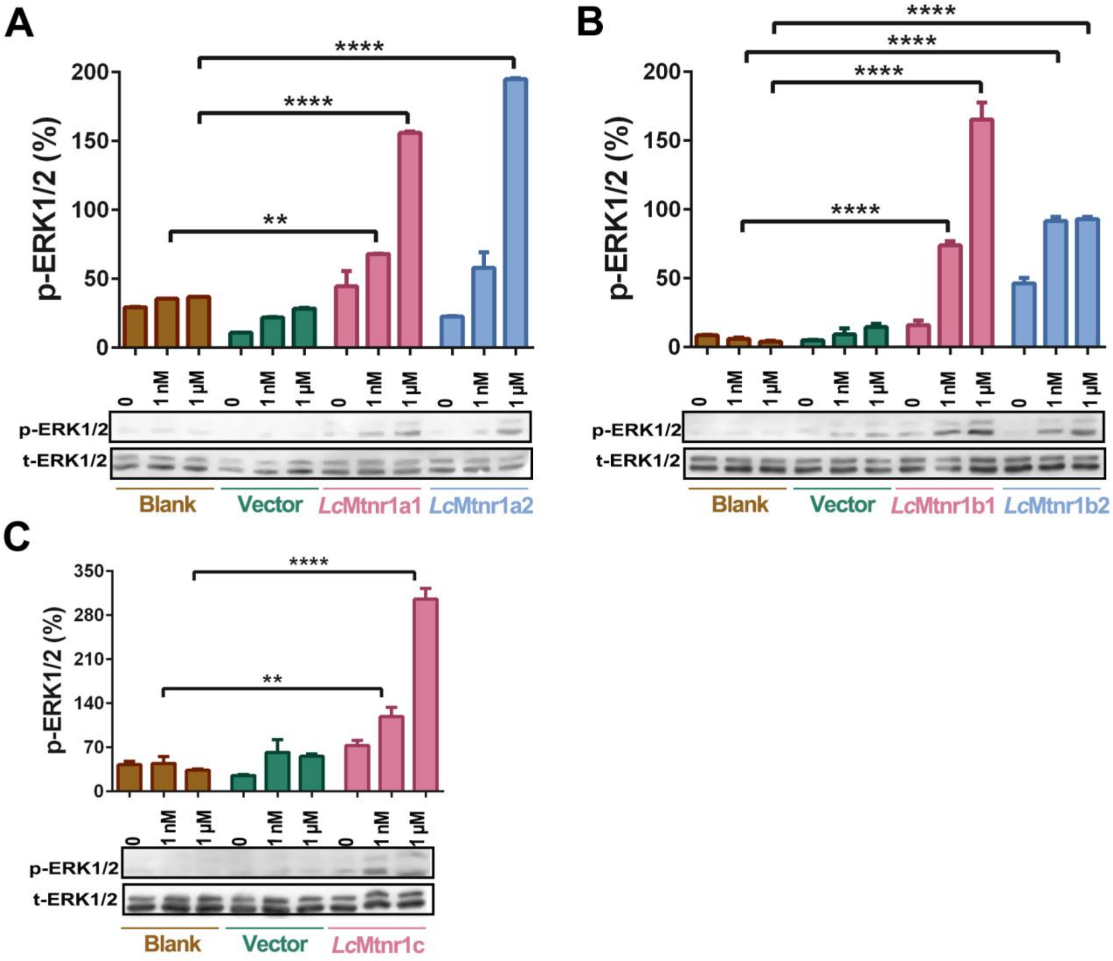

Figure S11. MT-induced ERK1/2 phosphorylation in HEK293 cells. Blank HEK293 cells, HEK293 cells stably expressing pFLAG-CMV-3 empty vector, *Lc*Mtnr1a1/2-FLAG (A), *Lc*Mtnr1b1/2-FLAG (B), and *Lc*Mtnr1c-FLAG (C) were stimulated with or without MT (1 nM and 1 μM) for 5 min, respectively. Data were analyzed using the one-way ANOVA followed by Tukey's multiple comparison test, ** $P < 0.01$, **** $P < 0.0001$. ERK1/2: extracellular regulated protein kinases 1/2.

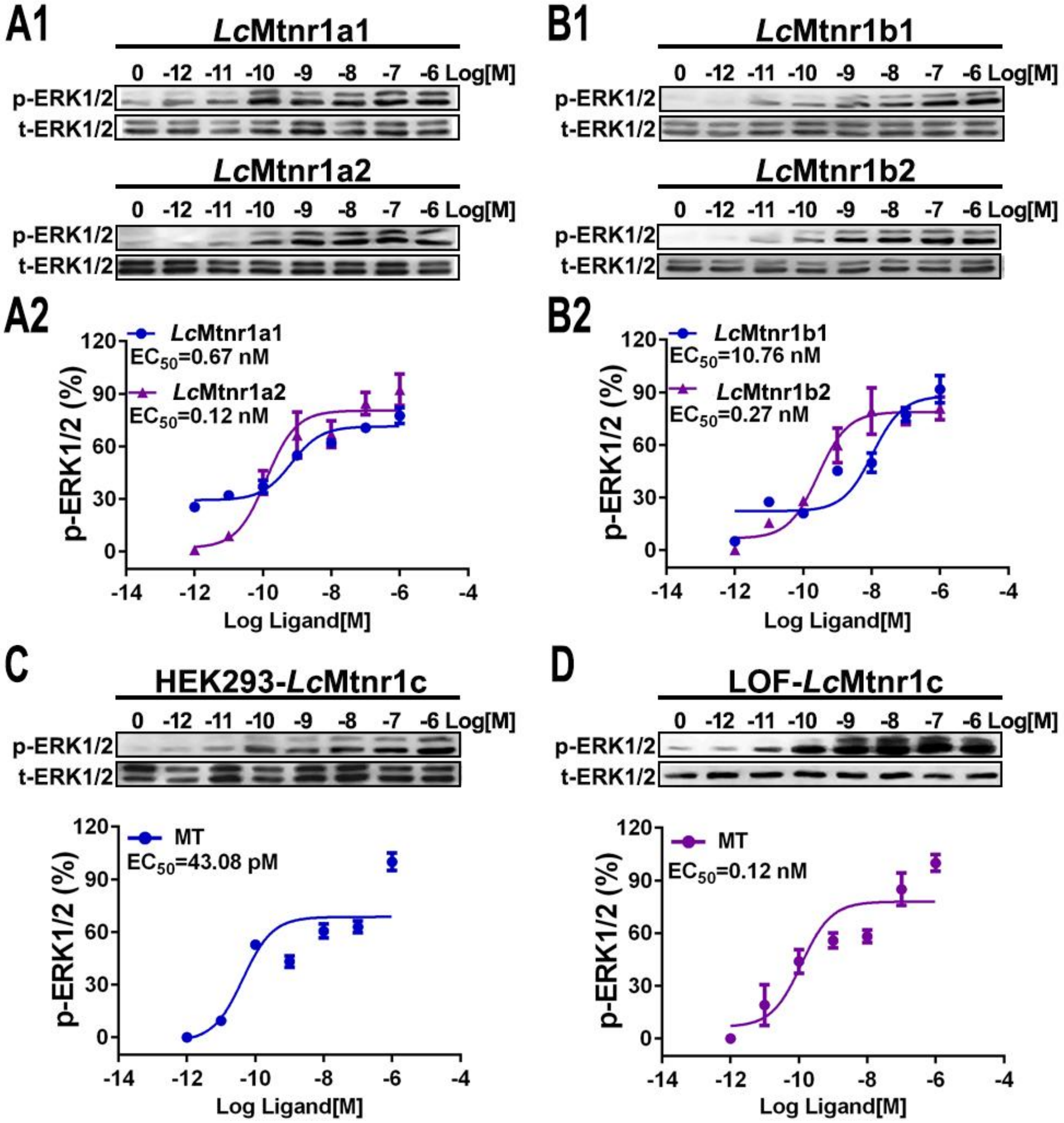

Figure S12. Concentration-dependent effects of MT on ERK1/2 phosphorylation in FLAG-*Lc*Mtnrs expressing cells. (A-C) and in LOF cells stably expressing FLAG-*Lc*Mtnr1c (D). HEK293-FLAG-*Lc*Mtnrs and LOF-FLAG-*Lc*Mtnr1c cells were stimulated with the indicated concentrations of MT for 5 min. ERK1/2: extracellular regulated protein kinases 1/2; EC50: concentration for 50% of maximal effect.

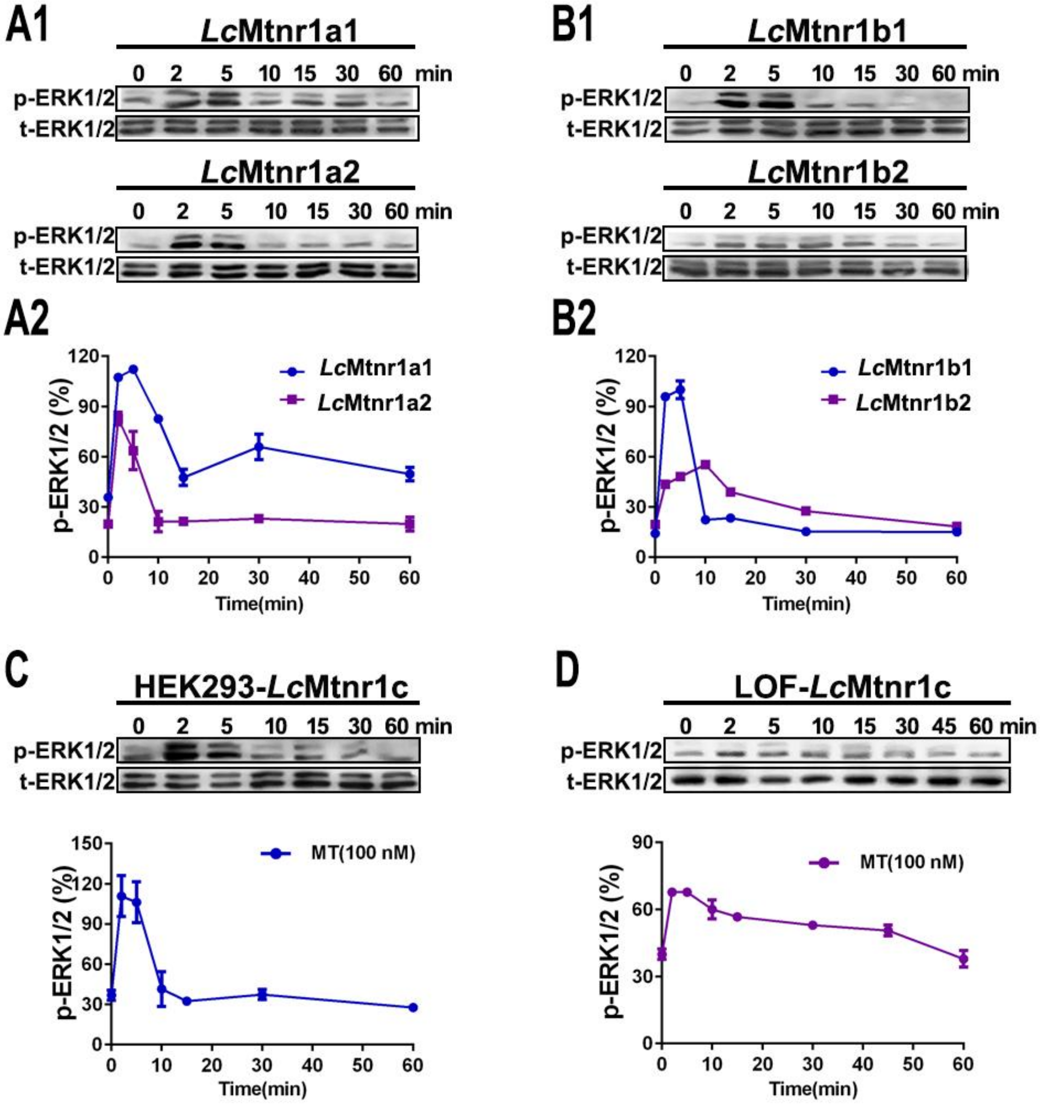

Figure S13. Time-dependent effects of MT on ERK1/2 phosphorylation in FLAG-*Lc*Mtnrs expressing HEK293 cells (A-C) and in LOF cells (D). HEK293-FLAG-*Lc*Mtnrs and LOF-FLAG-*Lc*Mtnr1c cells were stimulated with the 100 nM MT for the indicated periods of time. MT: melatonin; ERK1/2: extracellular regulated protein kinases 1/2.

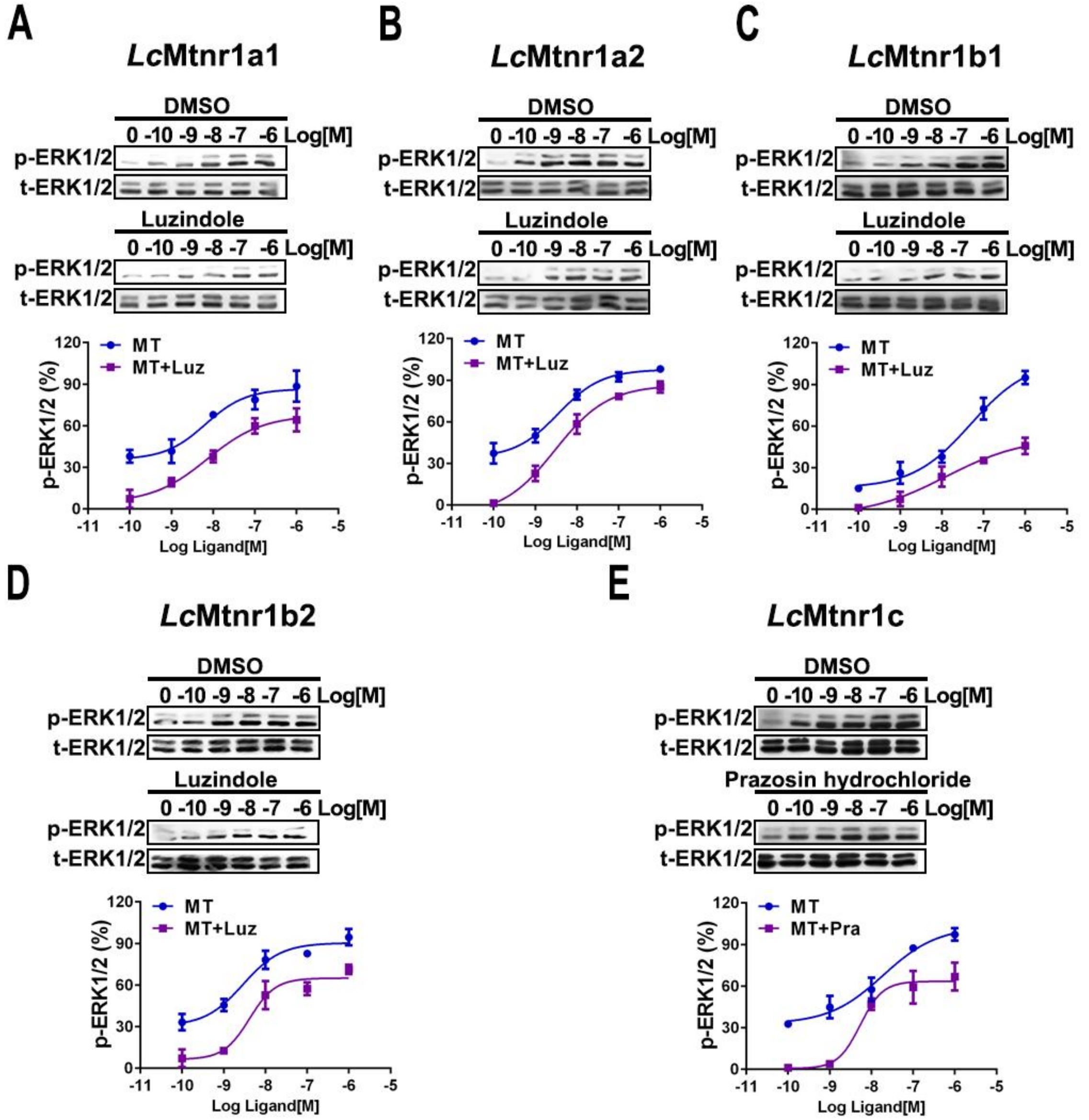

Figure S14. The *Lc*Mtnrs-mediated ERK1/2 phosphorylation activation inhibited by Mtnrs antagonists. HEK293-*Lc*Mtnr1a1/1a2/1b1/1b2 cells were pre-treated with vehicle (DMSO) or Mtnr1a/1b antagonists (10 µM Luzindole, Luz, 1 h) (A-D), or HEK293-*Lc*Mtnr1c cells were pre-treated with Mtnr1c antagonists (0.1 µM prazosin hydrochloride, Pra, 1 h) (E), and the cells were stimulated with the indicated concentration of MT for 5 min. MT: melatonin; ERK1/2: extracellular regulated protein kinases 1/2.

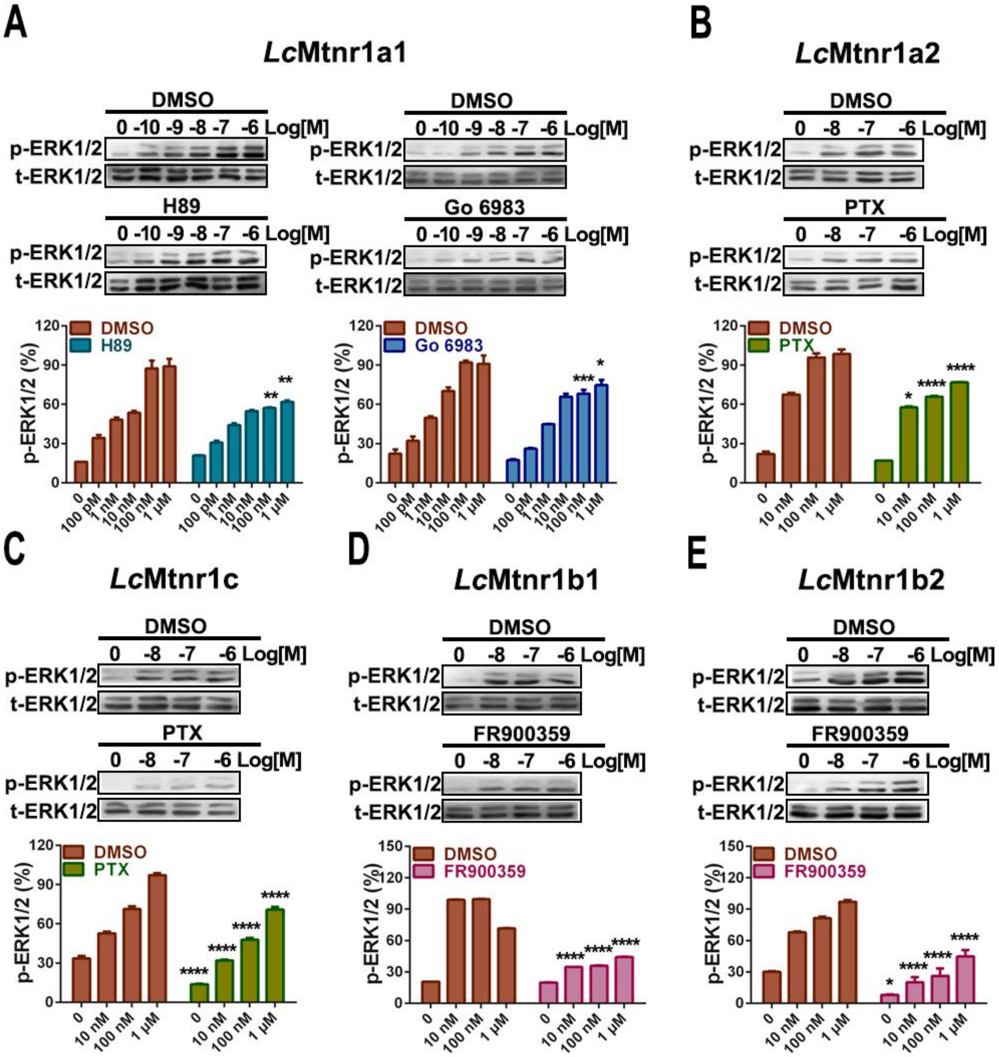

Figure S15. ERK1/2 activation mediated by *Larimichthys crocea* melatonin receptors. *(A)* ERK1/2 phosphorylation activated by *Lc*Mtnr1a1 was blocked by PKA or PKC inhibitors. HEK293-*Lc*Mtnr1a1 cells were pre-treated with DMSO, PKA inhibitor (H89, 10 μM), or PKC inhibitor (Go 6983, 10 mM) for 1 h prior to MT stimulation. *(B-E)* MT-induced (10 nM-1 μM, 5 min) ERK1/2 phosphorylation in *Lc*Mtnr1a2- expressing *(B)*, *Lc*Mtnr1c- expressing *(C)*, *Lc*Mtnr1b1-expressing *(D)*, and *Lc*Mtnr1b2-expressing *(E)* cells, in the absence or presence of PTX (50 ng/mL, 16 h) or FR900359 (1 μM, 1 h). Data are presented as means ± SEM. Data were analyzed using one-way ANOVA followed by Tukey's multiple comparison test (**P* < 0.05, ** *P* < 0.01, *** *P* < 0.001, **** *P* < 0.0001, all represent the difference between DMSO or inhibitor treated groups). PTX: pertussis toxin; PKA: Protein Kinase A; PKC: Protein Kinase C; H89: a PKA inhibitor; Go 6983: a PKC inhibitor; PTX: pertussis toxin; FR900359: an inhibitor of $G_{\alpha q}$; ERK1/2: extracellular regulated protein kinases 1/2; DMSO: dimethyl sulfoxide.

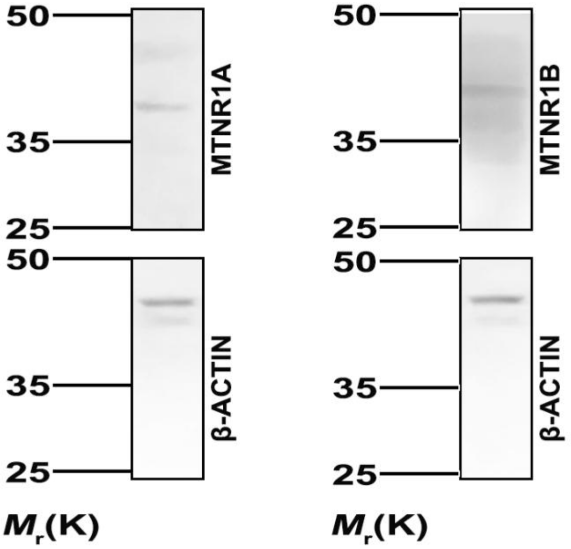

Figure S16. Immunoblot analysis of the antibody specificities of anti-Mtnr1a and anti-Mtnr1b IgG polyclonal antibodies in hypothalamus of large yellow croaker.

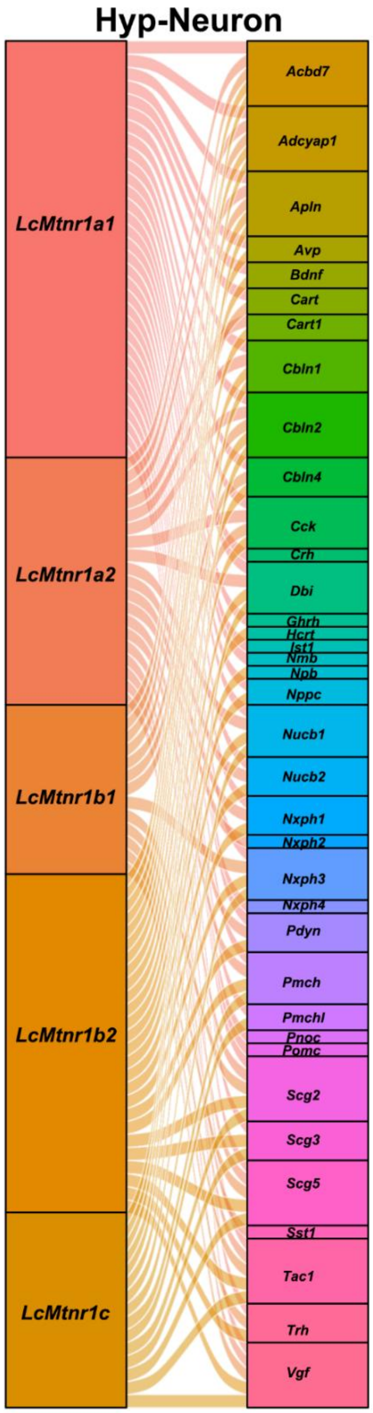

Figure S17. Co-expression of *Lc*Mtnrs and neuropeptides. Co-expression of *Lc*Mtnrs and neuropeptides (*Acbd7*, *Adcyap1*, *Apln*, *Avp*, *Bdnf*, *Cart*, *Cbln1*, *Cbln2*, *Cbln4*, *Cck*, *Crh*, *Dbi*, *Ghrh*, *Ist1*, *Nmb*, *Npb*, *Nppc*, *Nucb1*, *Nucb2*, *Nxph1*, *Nxph2*, *Nxph3*, *Nxph4*, *Pdyn*, *Pmch*, *Pmchl*, *Pnoc*, *Pmoc*, *Scg2*, *Scg3*, *Scg5*, *Sst1*, *Tac1*, *Trh* and *Vgf*) in hypothalamus neurons of large yellow croaker. The neuropeptide genes, detected with more than four reads per cell (reads ≥ 4), are given for further analyses. The diagram was generated by the Xiantao web (https://www.xiantao.love). Source data 1 lists the details. *Acbd7: Acyl-CoA binding domain*

containing 7; Adcyap1: adenylate cyclase activating polypeptide 1; Apln: apelin; Avp arginine vasopressin; Bdnf: brain derived neurotrophic factor; Cart: cocaine- and amphetamine-regulated transcript; Cbln1: cerebellin 1; Cbln2: cerebellin 2; Cbln4: cerebellin 4; Cck: cholecystokinin; Crh: corticotropin releasing hormone; Dbi: diazepam binding inhibitor, acyl-CoA binding protein; Gnrh: growth hormone releasing hormone; Hcrt: hypocretin receptor; Ist1: increased sodium tolerance 1 homolog; Nmb: neuromedin B; Npb: neuropeptide B; Nppc: natriuretic peptide C; Nucb1: nucleobindin 1; Nucb2: nucleobindin 2; Nxph1: neurexophilin 1; Nxph2: neurexophilin 2; Nxph3: neurexophilin 3; Nxph4: neurexophilin 4; Pdyn: prodynorphin; Pmch: pro-melanin concentrating hormone; Pmchl: pro-melanin-concentrating hormone, like; Pnoc: prepronociceptin; Pomc: proopiomelanocortin; Scg2: secretogranin II; Scg3: secretogranin III; Scg5: secretogranin V; Sst1: susceptibility to tuberculosis 1; Tac1: tachykinin precursor 1;Trh: thyrotropin releasing hormone; Vgf: VGF nerve growth factor inducible.

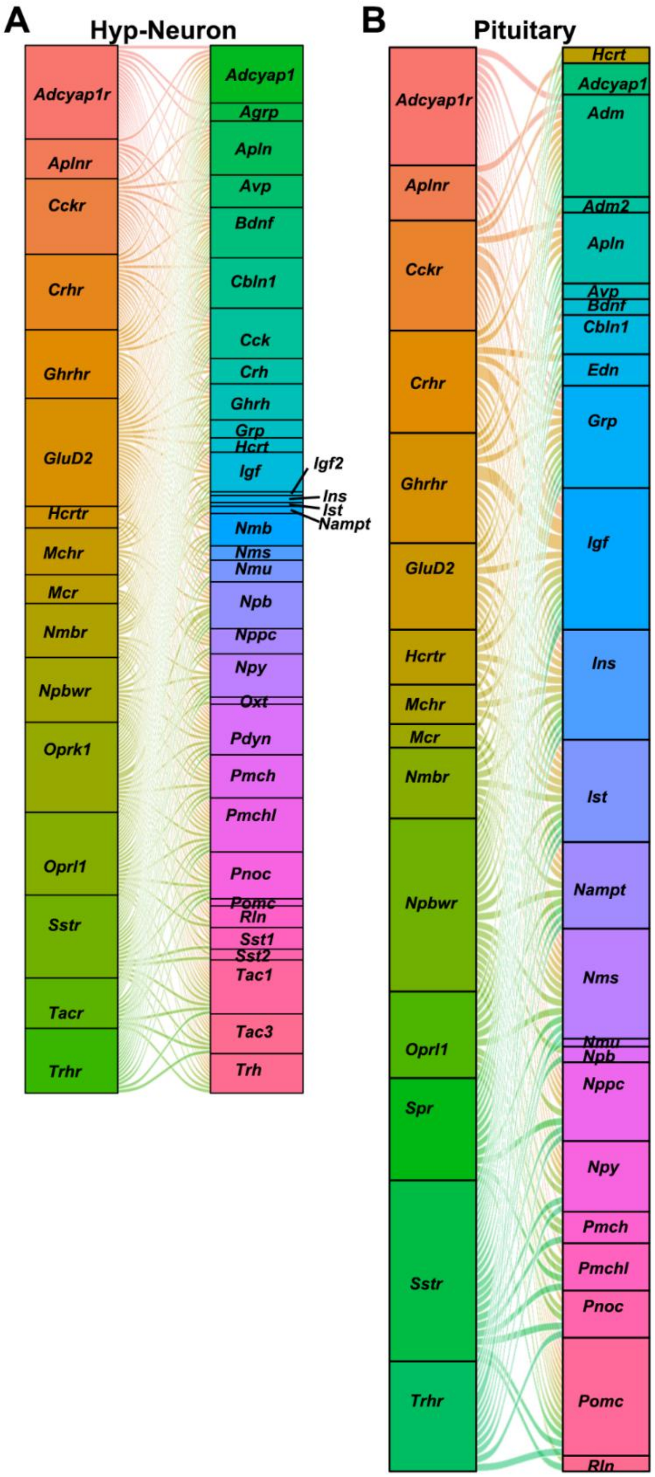

Figure S18. Co-expression of neuropeptides' receptors and other neuropeptides in hypothalamus neurons or pituitary cells. The expression cells of the neuropeptide receptors in hypothalamus neurons (A) or pituitary cells (B) were screened corresponding to the neuropeptides shown in Figure S16. The diagram was generated by the Xiantao web (https://www.xiantao.love). Source data 2 and 3 list the details. *Adcyap1r: pituitary adenylate*

cyclase-activating polypeptide type I receptor; Aplnr: apelin receptor; Cckr: cholecystokinin receptor; Crhr: corticotropin-releasing hormone receptor; Gnrhr: gonadotropin releasing hormone receptor; GluD2: glutamate dehydrogenase 2; Hcrtr: hypocretin receptor; Mchr: melanin concentrating hormone receptor; Mcr: mast cell regulator; Nmbr: neuromedin B receptor; Npbwr: neuropeptides B and W receptor; Oprk1: opioid receptor kappa 1; Oprl1: opioid related nociceptin receptor 1; Sstr: somatostatin receptor; Tacr: tachykinin peptides receptor; Trhr: thyrotropin releasing hormone receptor; Adcyap1: adenylate cyclase activating polypeptide 1; Agrp: agouti related neuropeptide; Apln: apelin; Avp: arginine vasopressin; Bdnf: brain derived neurotrophic factor; Cbln1: cerebellin 1; Cck: cholecystokinin; Crh: corticotropin releasing hormone; Gnrh: growth hormone releasing hormone; Hcrt: hypocretin receptor; Igf: insulin-like growth factor;Igf2: insulin like growth factor 2; Ins: insulin; Ist: IST1 factor associated with ESCRT-III; Nampt: nicotinamide phosphoribosyltransferase; Nmb: neuromedin B; Nms: neuromedin S; Nmu: neuromedin U; Npb: neuropeptide B; Nppc: natriuretic peptide C; Npy: neuropeptide Y; Oxt: oxytocin/neurophysin I prepropeptide; Pdyn: prodynorphin; Pmch: pro-melanin concentrating hormone; Pmchl: pro-melanin-concentrating hormone, like; Pnoc: prepronociceptin; Pomc: proopiomelanocortin; Rln: relaxin; Sst1: susceptibility to tuberculosis 1; Sst2: susceptibility to tuberculosis 2; Tac1: tachykinin precursor 1; Tac3: tachykinin precursor 3; Trh: thyrotropin releasing hormone; Spr: sepiapterin reductase; Adm: adrenomedullin; Adm2: adrenomedullin 2; Grp: gastrin releasing peptide.

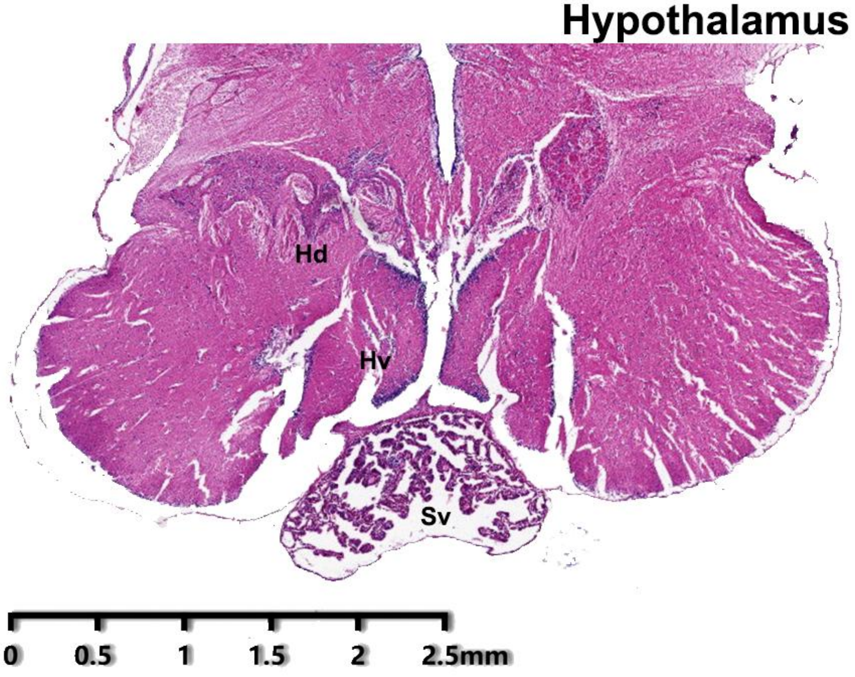

Figure S19. Microscopy of hematoxylin-eosin stained section of large yellow croaker hypothalamus. Hv: the ventral zones of the hypothalamus; Hd: the dorsal zones of the hypothalamus; Sv: Saccus vasulosus. Scale, as indicated.

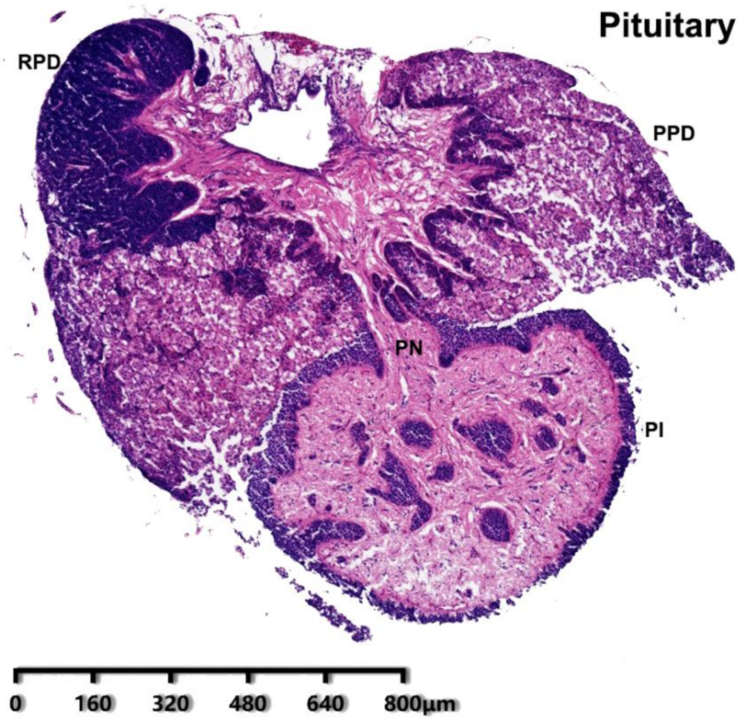

Figure S20. Microscopy of hematoxylin-eosin stained section of large yellow croaker pituitary. RPD: rostral pars distalis; PPD: proximal pars distalis; PI: pars intermedia; PN: neurohypophysis. Scale, as indicated.

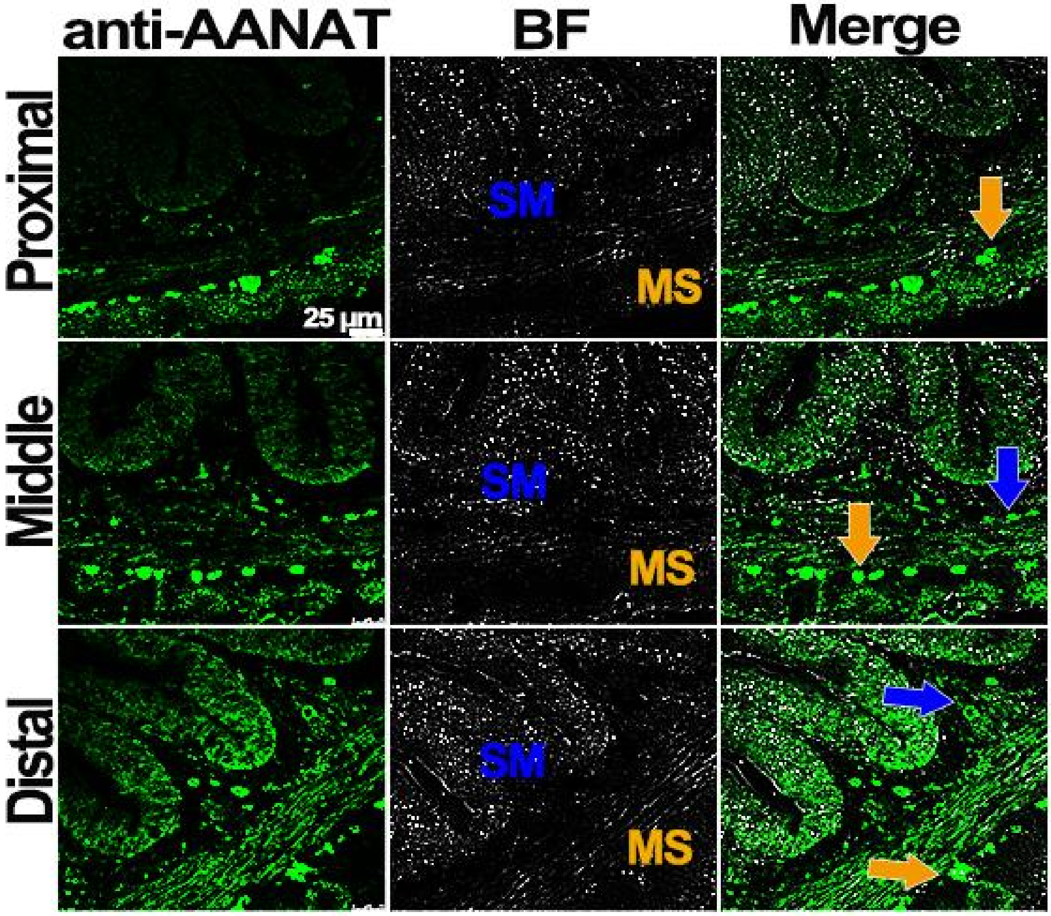

Figure S21. Distribution of AANAT in intestine of large yellow croaker. Immunohistochemical images of presumed MT-synthesizing cells in the intestine of large yellow croaker. The "AANAT" refers to the intestinal sections which were incubated with the anti-AANAT antibody. BF: Bright Field. Arrowheads in merged images indicate $AANAT^{+}$ cells. Ms: muscularis; Sm: submucosa.

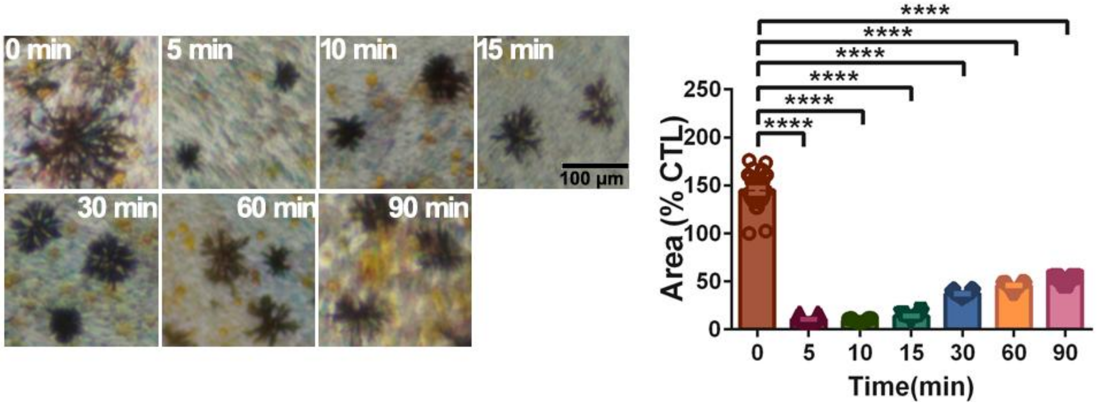

Figure S22. MT-mediated regulation of melanophore mobilization in the large yellow croaker *in vivo*. The bar chart shows the areas of melanophore covered the dorsal skin after indicated times treatment with 5.0 nM MT. The results are expressed as mean (± SEM) and are expressed as areas of melanophores relative to nontreated groups. Data were analyzed using the

one-way ANOVA followed by Tukey's multiple comparison test, **** $P < 0.0001$.

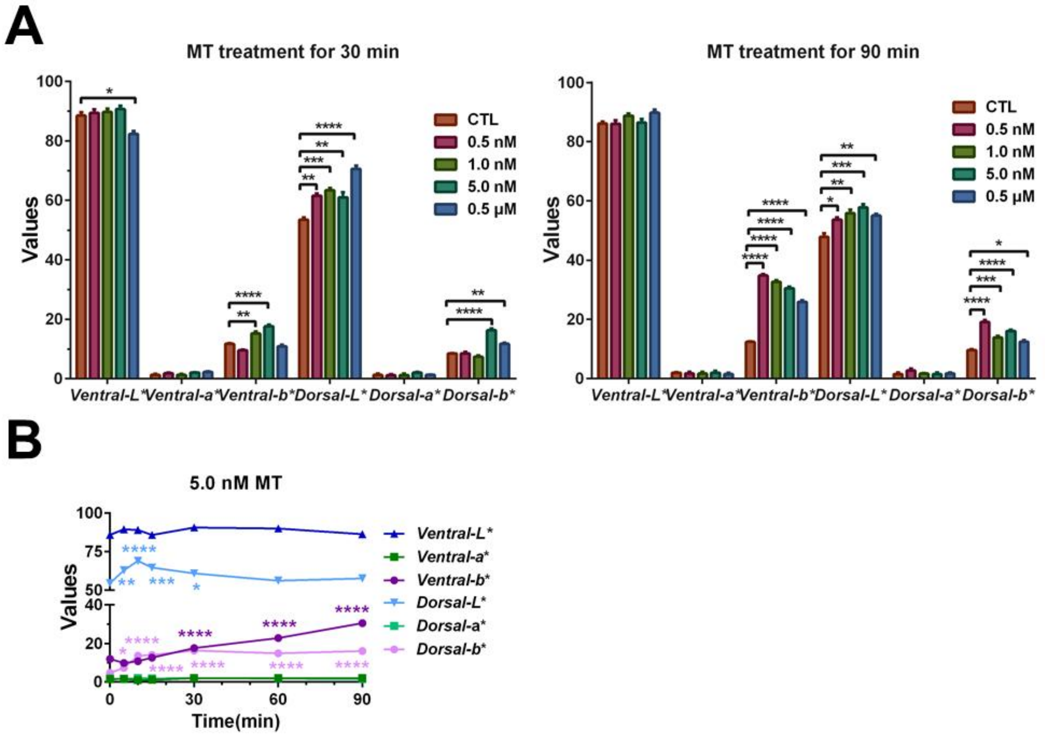

Figure 23. Effects of MT on the skin colors of the adult large yellow croaker. The L^*, a^*, and b^* values were measured for the ventral and dorsal treated by different concentrations of MT for 30 min and 90 min (A) as well as 5.0 nM MT treated within 90 min (B), respectively. Each data represents mean (± SEM) (n=3). Data were analyzed using the one-way ANOVA followed by Tukey's multiple comparison test, *$P < 0.05$, ** $P < 0.01$, *** $P < 0.001$, **** $P < 0.0001$. MT: melatonin; CTL: control.

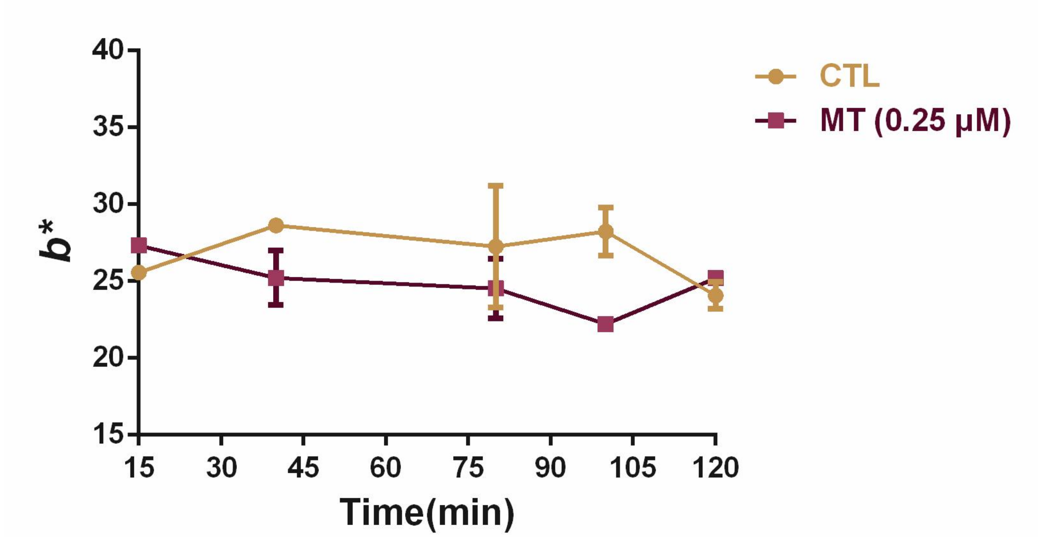

Figure S24. The b^* values from ventral skin of large yellow croaker with 0.25 μM MT treatment for indicated times *in vitro*. Data were analyzed using the two-way ANOVA

followed by Sidak's multiple comparison test. MT: melatonin.

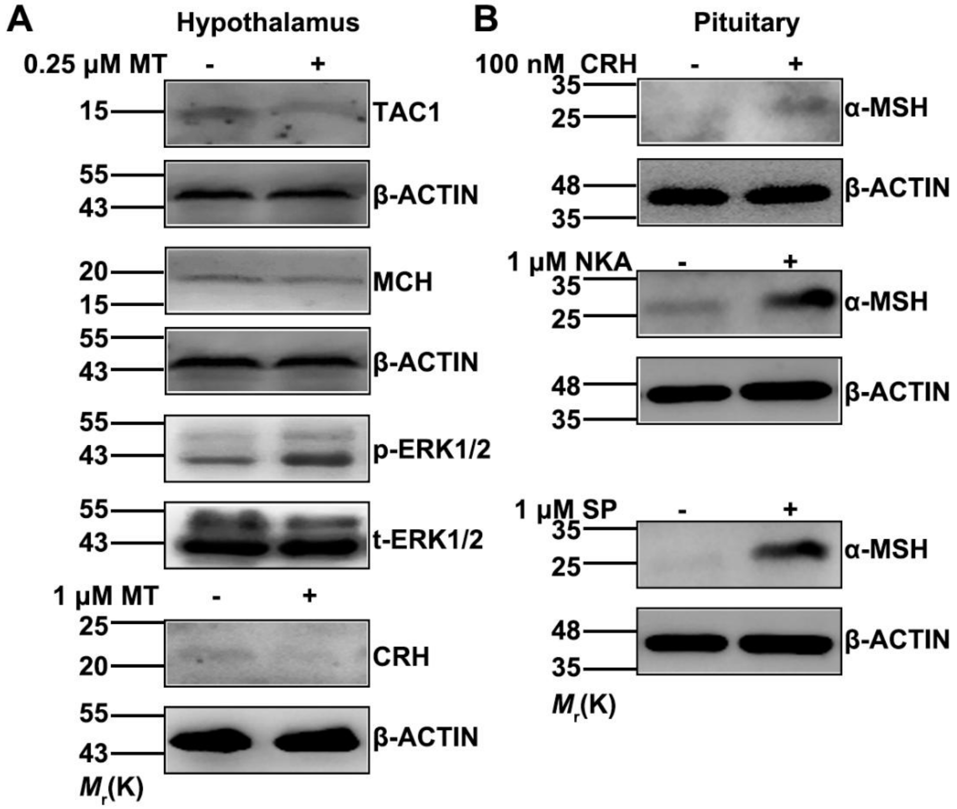

Figure S25. Immunoblot analysis of TAC1, MCH and p-ERK1/2 in hypothalamus and α-MSH in pituitary of large yellow croaker. (A) Samples were collected and lysed after 30 min of MT (0.25 μM and 1 μM) stimulation. (B) Samples were collected and lysed after 30 min of 100 nM CRH, 1 μM neurokinin A (NKA), and 1 μM substance P (SP) stimulation, respectively. The amino acid sequences of CRH, NKA and SP are as follows: CRH, SEDPPISLDLTFHLLREMMEMSRAEQLAQQAQNNRRMMELF-NH_2; NKA, HKVNSFVGLM-NH_2; SP, KPRPHQFIGLM-NH_2. MT: melatonin; TAC1: Tachykinin Precursor 1; MCH: melanin-concentrating hormone; CRH: corticotropin releasing hormone; α-MSH: α-melanocyte-stimulating hormone (MSH); ERK1/2: Extracellular signal-Regulated Kinase 1/2.

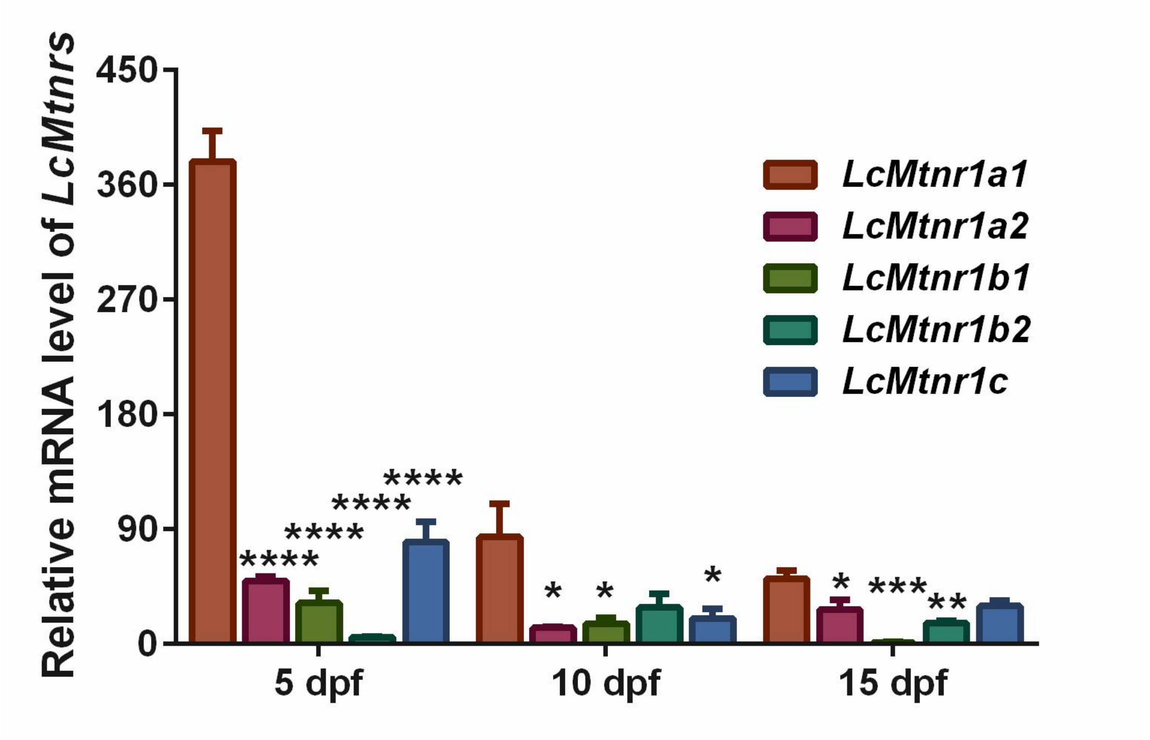

Figure S26. Relative expression of *Lc*Mtnrs mRNA at different developmental stages of large yellow croaker larvae. Each symbol and vertical bar represent mean ± SED (n = 6). *P < 0.05, ** P < 0.01, *** P < 0.001, **** P < 0.0001 compared with *Lc*Mtnr1a1, by one-way analysis of variance (ANOVA) using Tukey's multiple comparison test. dpf: day post fertilization.

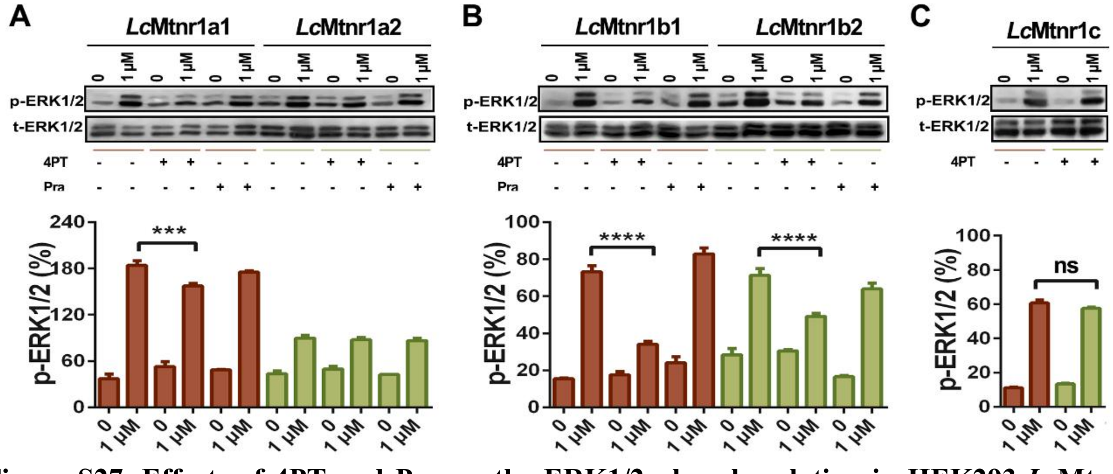

Figure S27. Effects of 4PT and Pra on the ERK1/2 phosphorylation in HEK293-*Lc*Mtnrs cells. Samples were measured after 5 min of 1 μM MT stimulation with or without 1 h of 4-P-PDOT (4PT) (0.1 μM) or , 50 μM prazosin hydrochloride (Pra) (0.1 μM) pre-treatment. Data were analyzed using the one-way ANOVA followed by Tukey's multiple comparison test, *** P < 0.001, **** P < 0.0001. ns, not significant. ERK1/2: Extracellular signal-Regulated Kinase 1/2.

Table S1.

Accession numbers of melatonin receptor protein sequences of different species were used for phylogenetic analysis and alignment.

Species	Gene synonym	Accession no.
Homo sapiens	Mtnr1a	NP_005949.1
Mus musculus		NP_032665.1
Danio rerio		NP_571468.1
Takifugu alboplumbeus		BAI39598.1
Gallus gallus		NP_990693.1
Dicentrarchus labrax		ACB13280.1
Epinephelus coioides		AGO03817.1
Esox lucius		XP_010889900.1
Carassius auratus		BAI65861.1
Lepisosteus oculatus		XP_006631980.2
Salmo salar		XP_014064448.1
Salmo salar		XP_014050730.1
Salmo salar		XP_014068290.1
Salmo salar		XP_014068723.1
Siganus guttatus		ABG77572.1
Homo sapiens	Mtnr1b	NP_005950.1
Mus musculus		NP_663758.2
Gallus gallus		NP_001280032.1
Danio rerio		NP_571469.1
Takifugu alboplumbeus		BAI39599.1
Dicentrarchus labrax		ACB13281.1
Epinephelus coioides		AGO03818.1
Esox lucius		NP_001290778.1
Carassius auratus		BAI65864.1
Lepisosteus oculatus		XP_015196706.1
Salmo salar		XP_014070615.1
Gallus gallus	Mtnr1c	NP_990692.1
Xenopus laevis		NP_001081388.1
Danio rerio		NP_001154956.1
Carassius auratus		BAI65865.1
Dicentrarchus labrax		ACB13282.1
Takifugu alboplumbeus		BAI39600.1
Siganus guttatus		ABG77573.1
Epinephelus coioides		AGO03819.1
Esox lucius		XP_010896763.1
Homo sapiens	5-HT1R	BAA94488.1
Homo sapiens		BAA01763.1
Mus musculus		EDL18479.1
Danio rerio		NP_001116793.1
Danio rerio		NP_001139238.1
Gallus gallus		NP_001163999.1
Xenopus laevis		NP_001079299.1
Salmo salar		XP_013999610.1
Homo sapiens	5-HT2R	EAX08770.1
Mus musculus		EDL35846.1
Danio rerio		ABI18978.1
Gallus gallus		AEV42168.1
Larimichthys crocea		KAE8279357.1

Homo sapiens	5-HT4R	CAN84676.1
Mus musculus		CAA09598.1
Larimichthys crocea		KAE8284925.1
Salmo salar		XP_014055545.1
Homo sapiens	5-HT5R	EAX04526.1
Mus musculus		EDL37224.1
Mus musculus		EDL39785.1
Salmo salar		XP_014036159.1
Homo sapiens	5-HT6R	AAA92622.1
Mus musculus		AAD46490.1
Larimichthys crocea		KAE8293148.1
Homo sapiens	5-HT7R	AAC37538.1
Mus musculus		EDL41763.1
Danio rerio		XP_690599.7
Xenopus laevis		NP_001079253.1
Larimichthys crocea		KAE8280972.1
Salmo salar		XP_014026341.1

Table S2.

Sequences of primers used for vector construction, qPCR analysis, and sequence identification for *LcMtnrs*.

Primer	Sequence (5′-3′)	Application	Identifier
Lcmtnr1a1-seq-F	CCCAAGCTTATGGTTATAAATGGGTC	vector construction	NCBI BioProject, PRJNA354443
Lcmtnr1a1-seq-R	CGGGATCCCAGACAGAGTCCACC		
Lcmtnr1a2-seq-F	CCCAAGCTTATGCTGAATGGACCG	Sequence identification	GenBank: OM939679
Lcmtnr1a2-seq-R	GGGGTACCCAGCAGTGAAACTTG		
Lcmtnr1b1-seq-F	CCAAGCTTATGCCGGACACATTC		GenBank: OM939680
Lcmtnr1b1-seq-R	GGGGTACCCATTCTTTGTTTGTG		
Lcmtnr1b2-seq-F	CCAAGCTTATGTCCATGCAAGAAG		NCBI BioProject, PRJNA354443
Lcmtnr1b2-seq-R	CGCGGATCCCACAGAGTCTCTGC		
Lcmtnr1c-seq-F	CCAAGCTTATGGATTTAGAGGTG		GenBank: OM939678
Lcmtnr1c-seq-R	GGGGTACCGATACATTTATCTCTGC		
β-actin F	TCGTCGGTCGTCCCAGGCATCAG	Internal control	GenBank: EU443733.1
β-actin R	ATGGCGTGGGGCAGAGCGTAACC		
Lcmtnr1a1-q-F	TACAGGTGAGGAGACGAGTGAAG	qPCR	NCBI BioProject, PRJNA354443
Lcmtnr1a1-q-R	GAAGTAGGCCATGAAGTAGCTGG		
Lcmtnr1a2-q-F	GTGAGCTCGCTGTACACTATCAC		GenBank: OM939679
Lcmtnr1a2-q-R	CGACGAACATGGTGAGGAAGTTG		
Lcmtnr1b1-q-F	ACACAGTGGCAGTAGTAGTGGT		GenBank: OM939680
Lcmtnr1b1-q-R	CAGCACAAAGACCACGAACATG		
Lcmtnr1b2-q-F	TCAACCGCTACTGTTACATCTGC		NCBI BioProject, PRJNA354443
Lcmtnr1b2-q-R	GTATGAGGTGCTGACTGTCTGTG		
Lcmtnr1c-q-F	ACATCTTCGTGGTGAGTTTGTCC		GenBank: OM939678
Lcmtnr1c-q-R	GTGATGTTGAAGATGGAGCCGAT		

Table S3.

The sources of *Lcmtnrs* probes, hairpins, and antibodies used for *In situ* HCR and immunofluorescence detection.

Reagent type	Designation	Source	Identifiers
Probe	*Lcmtnr1a1*-B4 (RefSeq ID: XP_010727592.1)	Molecular Instruments	LOT.PRH057
Probe	*Lcmtnr1a2*-B1 (RefSeq ID: XP_010741227.1)	Molecular Instruments	LOT.PRH054
Probe	*Lcmtnr1b1*-B3 (RefSeq ID: XP_027129075.1)	Molecular Instruments	LOT.PRH056
Probe	*Lcmtnr1c*-B2 (RefSeq ID: XP_010750373.1)	Molecular Instruments	LOT.PRH055
Hairpin/Fluorophore	B1h1 / Alexa514	Molecular Instruments	LOT.S031921
Hairpin/Fluorophore	B1h2 / Alexa514	Molecular Instruments	LOT.S032021
Hairpin/Fluorophore	B2h1 / Alexa488	Molecular Instruments	LOT.S035322
Hairpin/Fluorophore	B2h2 / Alexa488	Molecular Instruments	LOT.S034422
Hairpin/Fluorophore	B3h1 / Alexa594	Molecular Instruments	LOT.S029722
Hairpin/Fluorophore	B3h2 / Alexa594	Molecular Instruments	LOT.S031822
Hairpin/Fluorophore	B4h1 / Alexa647	Molecular Instruments	LOT.S034722
Hairpin/Fluorophore	B4h2 / Alexa647	Molecular Instruments	LOT.S025122
Antibody	MTNR1A-anti-rabbit IgG (polyclonal)	Abcam	CAT#ab203038
Antibody	MTNR1B-anti-rabbit IgG (polyclonal)	Abcam	CAT#ab203346
Antibody	GAD-65/67-anti-mouse IgG (monoclonal)	Santa Cruz	CAT#sc-365180
Antibody	FITC-conjugated goat anti-mouse IgG (polyclonal, goat)	Beyotime	CAT#A0568
Antibody	Cy3-conjugated goat anti-rabbit IgG (polyclonal, goat)	Beyotime	CAT#A0516
Antibody	TAC1-anti-rabbit IgG (polyclonal)	Proteintech	CAT#28599-1-AP
Antibody	CRF-anti-rabbit IgG (polyclonal)	ThermoFisher	CAT#PA5-102356
Antibody	NPY-anti-rabbit IgG (polyclonal)	ThermoFisher	CAT#PA5-95226
Antibody	ACBD7-anti-rabbit IgG (polyclonal)	Sigma	CAT#HPA062478
Antibody	MCH-anti-rabbit IgG (polyclonal)	Sigma	CAT#M8440

Source data files:

Source data1. The hypothalamus peptidergic neurons co-expressing *LcMtnrs*.

Source data2. The gene expression of neuropeptide receptors in hypothalamus neurons.

Source data3. The gene expression of neuropeptide receptors in pituitary cells.
